# Divergent preference functions generate directional selection in a jumping spider

**DOI:** 10.1038/s41598-023-50241-x

**Published:** 2023-12-21

**Authors:** Leonardo Braga Castilho

**Affiliations:** Universidade de Brasília, Brasília, DF Brazil

**Keywords:** Behavioural ecology, Sexual selection

## Abstract

Sexual selection has long been thought to promote speciation, but this possibility still remains a topic of controversy. Many theoretical models have been developed to understand the relationship between sexual selection and speciation, but such relationship seems complex and sexual selection has also been argued to prevent speciation in many scenarios. Here, I model for the first time the tendency of speciation due to sexual selection using realistic model parameters input collected from an existing species, the jumping spider *Hasarius adansoni*. I show that, even though the species has substantial female variance in preference (the model typically thought to link sexual selection to speciation), when realistic parameters are input in the model, it predicts directional selection, rather than disruptive selection. I propose that including realistic parameters in speciation models is a new tool that will help us understand how common sexual selection helps or hinders speciation in the real world.

## Introduction

Sexual selection has long been known to have important evolutionary consequences, the most recognized being the evolution of extravagant secondary sexual characters, sexual displays, and animal weaponry^1^. Much less obvious, however, is the importance of sexual selection to the process of speciation. Darwin^2^ was the first to hypothesize that sexual selection could facilitate speciation, by driving species differentiation through mate choice. Since then, many empirical and theoretical studies have investigated the evidence in favor of such phenomenon. Surprisingly, little consensus has been achieved. The most likely reason for why such a large body of research has led to little definitive knowledge of the subject is the fact that humans can rarely witness the process of speciation, so evidence regarding such process must be largely correlational and indirect.

Lande^3^ was the first to model the speciation process through sexual selection. He found that, if there is enough variation in female preference along a cline, and if both female preference and male ornament are heritable, speciation is likely to occur. Following such seminal work, several other models have showed similar results, highlighting the role of sexual selection in speciation in allopatry^4,5^, and in sympatry^6–9^. Other models, with more restrictive assumptions, have highlighted the necessity of some very specific conditions for sexual selection to promote species divergence. In fact, some conditions are so specific, that makes one wonder how often they are found in the real world. For example, Servedio and Bürger^10^ and Servedio^11^ showed that, with gene flow, sexual selection only helps speciation if preferences are relatively weak. Even though weak preferences are technically possible, the general consensus is that sexual selection based on mate preference (specially when the targed phenotype is morphological) is usually strong^12,13^, which would imply strong preferences. Given this scenario, one might ask how common weak preferences exist in nature and if the aforementioned models are evidence in favor or against speciation being caused by sexual selection in natural populations.

The problem is further complicated by some contrasting results in the literature. Some examples of such contrast rely on the study of sexual selection and speciation in birds. Birds have been particularly studied, given that secondary sexual characters are usually conspicuous to humans (i.e. song and plumage) and their taxonomy is relatively well understood. This leads to some studies trying to find a correlation between the degree of sexual dimorphism in taxonomic groups (which is interpreted as a proxy for sexual selection strength) and the number of clades in those groups (interpreted as a proxy for speciation rate). Such studies, however, have found contrasting results, with some authors pointing toward a positive correlation (e.g.^14^), while others point the lack of a correlation (e.g.^15^). Part of such confusion might come from the fact that those studies probably over represent characters easily recognized by humans (e.g. regular colors as opposed to ultraviolet reflectance). Studies using number of clades also face the problem of not accounting for species extinction, which can strongly affect the number of clades in the group. This might be why speciation by sexual selection has been found to be more prominent in young groups, in which extinction has not yet played a strong role in the number of clades^16^.

It has also been proposed that sexual selection may produce or hinder speciation, depending on other ecological factors. For instance, mate choice copying has been proposed to promote speciation in certain conditions, but hinder speciation in others^17^. The type of secondary sexual character considered, the taxon^16^, and even the sex under study^18^, have all been shown to change the probability of finding a positive relationship between sexual selection and speciation. In fact, the small number of studies that tried to investigate such relationship in individual species, also found mixed results. Three spine sticklebacks (*Gasterosteus* spp.) represent one of the most recent species divergence known today, and such process has long been thought to have been guided by sexual selection^19^. A similar example has been described in another fish, the African cichlid *Pundamilia* spp.^20^. However, the opposite has been found in the wolf spider *Schizocosa crassipes*, in which sexual characters are more similar between populations than other characters, showing that sexual selection is probably preventing speciation, rather than causing it^21^. This all points to a scenario in which a definitive unifying theory explaining the relationship between sexual selection and speciation might depend on a series of variables that differ from one species to another. This would require a body of work focusing on different species at a time, to finally show us how often sexual selection drives speciation and how often it hinders it, as well as the conditions needed for each scenario to occur.

The advantage of studying lower taxons (e.g. species, instead of genuses or families) is that one can collect data on several aspects of the animal (e.g. recruiting rate, number of offspring, mating success, mating preference, etc.) and explicitly include such values in theoretical models. This would give a higher power to predict what evolutionary paths the species will take with time. Here I model future evolutionary paths of a species by adding realistic parameters in the model, and ask if sexual selection is facilitating or hindering speciation. The parameters were adjusted according to data obtained from the jumping spider *Hasarius adansoni* since vast information about this species’ sexual selection is available^22,23^. I show that, when considering realistic parameters in the models, the evolutionary tendency might be different from what some more general theoretical models may predict.

## Methods

### Model species

The jumping spider *H. adansoni* is an urban species commonly found in the tropics. Previews experiments I conducted with the species produced data about the species reproduction. Castilho et al.^23^ showed that *H. adansoni* has individual variation in mate preference (as defined by Jennions and Petrie^24^, the preference is open ended), with some females preferring large males, while others prefer small males. Such variation dictates mating probability (some females are more likely to mate with small males, while others are more likely to mate with large males) and does not depend on female size (there is no assortative mating). Once mated, females tend to reject further copulation attempts. Also, after mating, females have a 69.5% chance of laying eggs. The number of young produced per female is positively correlated with male size^22^, and males are not selective, and usually court and mate any female presented to them^25^.

An interesting detail about *H. adansoni*’s sexual selection process is that it is similar to what theoretical models predict as being necessary for speciation^6–9,26^. Such models predict that, if there is enough variation in female preference, such that some females prefer high values of a male trait, while others prefer low values of such trait, sexual selection will be disruptive. Such scenario is thought to generate speciation even in sympatry. However, such models are theoretical and do not include any real species parameters in them. Thus, if the levels of individual variation seen in nature are sufficient to generate speciation remains unknown, and how other variables might prevent speciation even in such facilitating scenario has never been explored. Including parameters extracted from studied species will make such models more realistic, and enhance our predictive ability about speciation through sexual selection. I, thus, used *H. adansoni* real parameters to model the species future evolutionary path, and asked if sexual selection is facilitating or hindering speciation.

### Model overview

I built quantitative genetics models in which preference and size were both genetically determined. Each of the two traits are determined by *L* loci, with 3 possible alleles. Such alleles are − 1, 0, and 1. In the size loci, an individual with only − 1 allelles would be the smallest one in the population, while one with only 1 allelles would be the largest. Other combinations would generate intermediate phenotypes. In the preference loci, − 1 codes for a higher preference for small males, while 1 codes for a higher preference for large males. Other allelles combinations would generate intermediate preference functions (females that do not highly prefer either large or small males, see Fig. 2 in Castilho et al.^23^). I ran the same analysis with *L* = 5 (5-genes model), and with *L* = 10 (10-genes model). To prevent unrealistically high genetic drift effects, I added a probability of mutation to each allele per generation (since the presence of mutation can inhibit the loss of allelles by drift). Each of these models had four variants. One had a random noise to animals’ size, to simulate environmental effects on locus expression, another had 2 overlapping generations, with animals meeting (and possibly copulating) with other animals from their parental generation (but never with an animal from the generation of their grandparents), another with both of these effects and yet another with neither of these effects. Thus, in total, I had 8 models (i.e. 5-genes model; 5-genes model + environment; 5-genes model + overlap; 5-genes model + environment + overlap; 10-genes model; 10-genes model + environment; 10-genes model + overlap; 10-genes model + environment + overlap). The number of overlapping generations was chosen based on previews knowledge of *H. adansoni* life history. In laboratory, animals usually survive for many months, approximately the time it takes for a young to grow into adulthood, so 2 overlapping generations seems reasonable to happen in nature, while more than 2 seems unlikely (personal observation).

Each model starts with one thousand males and one thousand females, with preference and size normally distributed. Females find males randomly, and once a pair is matched, females have to decide between mating or leaving. The probability of mating is described by male size and female’s individual preference function. That is, the more a male size matches a given female’s preference for size, the higher the probability of an encounter resulting in copulation. If a female does mate with a male, she will stop accepting further mating attempts, since mated females of *H. adansoni* will rarely accept new copulations^22^. If, however, a female leaves without mating, it can be matched again with any male in the population (including others that have courted her previously) and the process restarts. Once a female mates, she has a 69.5% probability of laying eggs, and once eggs are laid, the number of young is a function of male size, as described by Castilho et al.^23^. This same process is repeated across generations until one could tell if selection for preference and size was directional, stabilizing, or disruptive, by looking at the histograms of phenotype values. Only the latter case would lead to speciation in the long run.

### The mathematical model and calculations

First, I considered *L* = 5, with each locus having 3 possible alleles (5-genes model). Since animals were diploid, each animal had 20 alleles (2 for each of the 5 loci coding for size, and another 2 for each of the 5 loci coding for preference).

The genotypes for size and preference of the initial males and females were established by randomly selecting 20 alleles from A = {− 1, 0, 1}, with replacement. I then considered another model, in which *L* = 10 (10-genes model), with the same 3 possible alleles for each locus. In this model, 40 allelles were randomly selected from A for each phenotype, and each individual was made up of 40 alleles. In both of these models, animal size was defined as the sum of its alleles. For the 5-genes + environment model and 10-genes + environment models, spiders’ size were calculated by adding a random number extracted from a normal distribution to the spider’s genotype for size. Such normal distribution was N (0, 1.5) for the 5 genes + environment models, and N (0, 2.16) for the 10 genes + environment models. Such distributions were chosen as to make the genetic effect standard deviation about 1.5 higher than the environmental effect standard deviation. For every model, one thousand males and one thousand females were generated through this process. To assess the effect of initial genetic variation in the model outcome, I built the 5-genes model described above, but with individuals only carrying − 1 or + 1 alleles (two sympatric populations with fixed alelles for size). However, such model resulted in the populations being rapidly mixed, and the final results were qualitatively the same as the other models, thus such result is not shown here.

After determining the genotypes of each individual, each female was given a probability of mating described by a realistic preference function. To achieve this, I used the data from Castilho et al.^23^. The authors paired each female with three different sized males and measured their propensity to copulate through a mixed binomial model, with female identity as the random factor. The authors found that the propensity to copulate (likelihood of copulating with an average male^27^) varies between females, and depends on male size, while adding female size to the model is necessary to keep random effects normality (Fig. S1A). I used the same data set with male and female sizes converted to z scores. I, then, built the same models as the authors, and found that the random intercept (α) had a mean µ_α_ ≈ 0.95, and a variance of σ^2^α ≈ 19.4. Random slopes of female identity on male size (β) had a mean µ_β_ ≈ 0.52, with a variance of σ^2^_β_ ≈ 5.7. Since female size was added as a fixed effect, the slope of every female for that effect was equal, with a value of − 2.9. The correlation between α and β was almost perfect^23^, and their covariance was Σ ≈ 10.4 (Fig. S1B).

For simplicity, I will describe in detail the creation of mating probabilities for the 5-genes and 5-genes + environment models, but the 10-genes and 10-genes + environment models follow the same logic. The models with overlapping generations only differ in the fact that animals can mate with other animals from the previous generation.

To create realistic values of female mating probabilities, I randomly extracted 1050 values from the multivariate normal distribution N_α,β_ (µ_α_, µ_β_, Σ_α,β_), where Σ_α,β_ is the 2 × 2 variance–covariance matrix of α and β. If a female had only − 1 alleles for preference (i.e. preference genotype equals − 10), her α and β values were randomly selected from the 50 lowest values of α and β extracted from N_α,β_. If her preference genotype was − 9, her α and β values were randomly selected from the 51th to 100th lowest values of α and β extracted from N_α,β_, and so on until reaching the 50th highest α and β values in N_α,β_. This ensured that females had phenotypes that matched their genotypes, while still adding some random noise to it.

It is also known that the number of young correlates with male size^23^. Using the data from Castilho et al.^23^, it is clear that the number of young a male sires can be approximated by a negative binomial distribution. Given the large mean and variance of the data, goodness of fit statistics might not work properly (Achim Zeileis, personal communication), but comparative histograms show a clear negative binomial distribution for number of young per male (Fig. S3).

The negative binomial distribution can be parameterized as:

1 $$ f\left( {y;k,\mu } \right) = \frac{{\Gamma \left( {y + k} \right)}}{{\Gamma \left( k \right) \times \Gamma \left( {y + 1} \right)}} \times \left( {\frac{k}{\mu + k}} \right)^{k} \times \left( {1 - \frac{k}{\mu + k}} \right)^{y} , $$

where parameter µ is the mean and k is the dispersion parameter, defined as $k = \frac{{\mu^{2} }}{{\sigma^{2} - \mu }}$, where σ^2^ is the distribution variance. Thus, there are two ways by which male size can affect number of young: it can affect a male’s µ, or both µ and k. To understand which of these processes occurs in *H. adansoni*, I accessed the raw data of Castilho et al.^23^, and was able to extract a total of 20 males for which data was available for size and total number of young after one single mating experiment (data available at^28^). First, I standardized all male and female sizes to z scores, and then I permuted all males in groups of three, with replacement, making up a total of 6,840 groups. For each group, I calculated mean male size (*M*), mean number of young (µ), variance of number of young (*σ*^2^), and *k*. By plotting mean male size with *µ* and *k*, it is possible to visualize how male size affects each of these parameters. The scatterplot between male size and *k* clearly shows that the variables are not related (Fig. S2C). Mean *k* was 3.8, with a very distinctive distribution and a few outliers. After removing outliers and negative values, I ended up with a set of realistic values for *k*, which will be called *K* (Fig. S2D). On the other hand, the scatter plot between male size and µ, shows an exponential positive relationship (Fig. S2A). When plotting only the raw data, consisting of the original 20 data points, the same exponential relationship appears (Fig. S2B). The relationship between male size and *µ* was then modeled from the permutation data set as $\mu = e^{a + bM + \varepsilon }$, where ε is the error. The equation estimated by the model was:

2 $$ \mu = e^{3.36 + 0.42M + \varepsilon } , \;\varepsilon \sim N\left( {0,\sigma = 13.15} \right) $$

With every model parameter specified, I ran the model for reproduction. Each one of the one thousand females were randomly paired with a male. When a female encountered a male, she had a probability of mating defined by:

3 $$ p\left( {copulation} \right) = \frac{{e^{{\alpha_{i} - 2.9F + \beta_{i} M}} }}{{1 + e^{{\alpha_{i} - 2.9F + \beta_{i} M}} }}, $$

where *α*_*i*_ and *β*_*i*_ represents, respectively, the intercept and slope of female *i* (as defined above), while *F* and *M* represent the z score for female and male size, respectively. If a female copulates, it has a 69.5% chance of laying eggs. And once a female copulates, it never copulates again. If a female does not copulate, it is paired again with a random male and the process restarts. If the female lay eggs, the number of young is defined as in Eq. (1), with each male’s *k* being a random sample from *K*, and *µ* defined as in Eq. (2).

Once a female laid eggs, the offspring genotype was determined as a free recombination of the parental alleles, and for each female, half of the young were assigned as males and the other half as females. The mutation rate was kept at 1% because it was enough to prevent genetic variability depletion, but still allowed a clear interpretation of how selection was acting on loci. The mutation consisted of substituting each allele by another, sampled with equal probability from the set *L* = {− 1, 0, + 1}, thus, a third of the mutations did not affect the final genotype (substituting the allele by itself).

The process of reproduction with mutation was repeated until animals’ size distribution became constant at each generation.. To avoid unrealistic computer processing times, every time the adult population of one sex was above 10,000, a number of random animals of that sex was killed and the population was kept to a maximum of 10,000 individuals of each sex. Since the resulting models did not show any sign of divergent selection (see “Results”), I slowly changed the model parameters of the 5-genes model to understand what conditions must be met for speciation to happen. First, I removed fecundity selection (i.e. number of young were no more dependent on male size), then I set all females’ *α*_*i*_ to − 5, which lead to a very small probability of mating with average sized males (Fig. S18A), and finally, I made females’ *βi* dependent on female size, leading to a assortative mating by size.

All simulations were performed in R 4.3.1 and the full code is available in the Supplementary Material.

## Results

**Figure 1:**
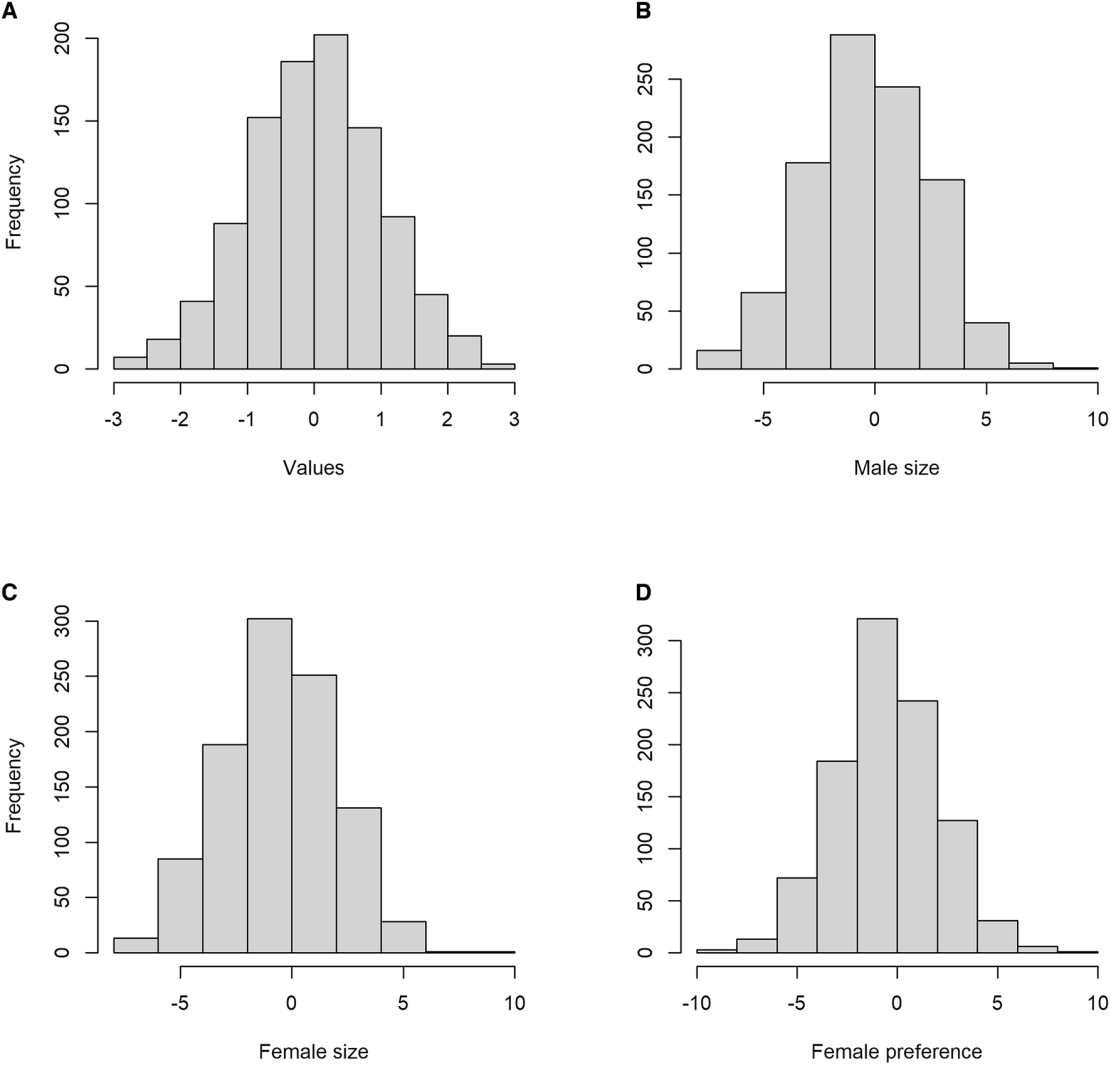
Initial values simulated for *Hasarius adansoni* phenotypes in the 5-genes model. (**a**) A random sample from a normal distribution, for comparison. (**b**) Male size. (**c**) Female size. (**d**) Female preference. In this simulation, each phenotype is controlled by 5 genes, each of which has 3 possible alleles.

**Figure 2:**
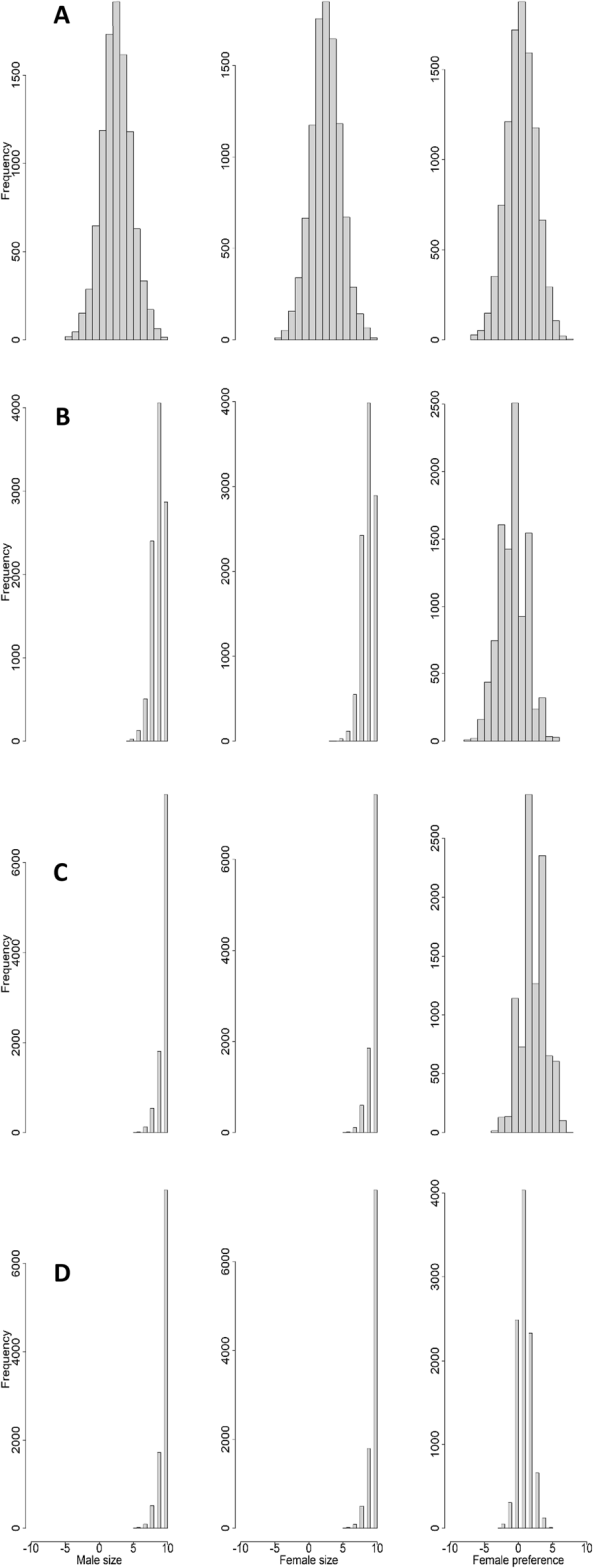
Evolutionary path of size and female preferences of *Hasarius adansoni* simulated by the 5-genes model. Figure shows frequency distributions of all phenotypes after (**a**) 5 generations, (**b**) 25 generations, (**c**) 45 generations, and (**d**) 75 generations. Parental frequency distributions are shown in Fig. 1. For similar simulations with different number of genes, with environmental influence on sizes and overlapping generations, see Supplementary Material.

In every model, initial values for all the phenotypes had normal distributions (Fig. 1, also see Supplementary Material), but male and female sizes were higher every generation, thus showing a clear directional selection. Preferences also reduced variability with generations, but not to the same extent as size, and the selection was not strictly directional, as the preference mean drifted to higher and lower values (Fig. 2, also see Supplementary Material). Adding environmental effect to animals’ size and/or overlapping generations did not change the results qualitatively and the outcome was essentially the same (Figs. S4–S9).

When I removed fecundity selection from the model, selection was not directional anymore, but still was not disruptive. In fact, such model resulted in stabilizing selection. Setting all *α*_*i*_ to − 5 forced mating choice to be extremely disruptive, with every female having very low probability of mating to an average sized male, and very high probability of mating with small or large males. Even though this produced some divergent selection at first, genetic drift quickly removed females with one of the two extremes mating choice groups (i.e. removed either females copulating with small males or those copulating with large males). With only females with one extreme preference phenotype present, selection was again directional, either selecting for larger or lower individuals, depending on what group of females genetic drift randomly removed. Only when I made females’ *β*_*i*_ dependent on female size, keeping *α*_*i*_ constant at -5 and removing fecundity selection, speciation finally happened (Fig. S18).

## Discussion

The models including realistic parameters performed very differently than similar theoretical models, with no realistic parameters. *H. adansoni* has a highly variable female preference for male size^23^. Such variable female preferences has long been thought by many authors to cause speciation, or at the very least, facilitate it^3,6–9,26^. However, in *H. adansoni* this very same scenario is obviously hindering speciation, since the trait under sexual selection studied is ultimately under directional selection, instead of a disruptive sexual selection that would lead to speciation. Regardless of the number of loci involved, the evolutionary outcome seems to be the same. Adding random variation in body size and/or overlapping generations did not change such outcome.

One question that rises from these results is why the trends observed here are directional, instead of divergent, as previous theoretical models suggest. In *H. adansoni*, larger males sire more offspring. This obviously poses a selection in favor of larger body size. However, even though removing such fecundity selection ended directional selection, it still did not cause disruptive section. It actually produced stabilizing selection. Since the number of animals with average size is larger than animals with extreme sizes, females preferring extreme male sizes were so rarely finding males of their preferred size, that they ended up meeting with average sized males. And since females only copulate once, this leaves most extreme sized males without contributing to the genetic pool of the next generation, and selection ends up stabilizing. I encourage researchers working with similar species, but in which females copulate many times throughout their lives, to produce similar models, and verify if speciation is more likely to occur in such scenario. As to the models shown here, disruptive selection (and consequently speciation) only happened when mate choice was strongly disruptive and dependent on female size, producing assortative mating. This forced the few females preferring extremed sized males that actually found them, to contribute to the gene pool of the next generation with their alleles coding small size, breaking the stabilizing selection process.

This study shows the importance of inputing realistic values in theoretical models. Throughout the years, numerous models were built, trying to predict the influence of sexual selection on speciation^3,4,7,8,10,29^. The general consensus seems to be that sexual selection can help speciation in some specific scenarios, but probably needs the help of ecological selection in most or all cases^30–32^. However, we do not know how common this happens in the evolutionary process, since sexual selection can also hinder speciation in many other scenarios (see^16^, and this study). It is, thus, difficult to make generalizations about the relationship between sexual selection and speciation. Here, I show evidence that, despite the disruptive sexual preferences in *H. adansoni*, the general sexual selection hinders speciation by being directional, instead of promoting it by being divergent, as many theoretical models suggest. Given the results shown, I also propose that sexual selection might have a higher probability of helping speciation in species where assortative mating is present.

It is important to note that it is usually impractical to have absolutely all relevant parameters to a model extracted directly from the species, as usually many factors can influence the outcome of the model (e.g. life expectancy, predation rate, female cryptic choice, sperm competition, etc.). Thus, a model can never be based in its entirety in realistic parameters. However, imputing as many realistic parameters as possible will result in more realistic models and help us understand what conditions facilitate or hinder speciation. Moreover, one important advantage of such approach is that the model can then be updated as new data from the species become available. Thus, building realistic models of different species with different niches, and updating them with new information, will help us finally understand how common sexual selection promotes speciation and what are the conditions for this to happen.

### Supplementary Information

Supplementary Information 1.

Supplementary Figures.

## Data Availability

All the relevant code used in this article is available as a supplementary material. The data used to extract the relationship between number of young, µ, and k, is available publicly at figshare.com.
