# Supplementary material for: Divergent preference functions generate directional selection in a jumping spider
Source: Sci Rep. 2023 Dec 21;13:22794. doi: 10.1038/s41598-023-50241-x (PMC10739821; doi:10.1038/s41598-023-50241-x)
Supplement: Supplementary file 1 — Supplementary Information 1. [file 41598_2023_50241_MOESM1_ESM.docx]

**Divergent preference functions generate directional selection in a jumping spider**

Behavioral Ecology and Sociobiology

Leonardo Braga Castilho

[leonardobcastilho@gmail.com](mailto:leonardobcastilho@gmail.com)

Code of the simulations performed.

#5 GENES FOR EACH PHENOTYPE

#CREATING PARENTAL POPULATION

m=numeric(0)

f=numeric(0)

popM<-list()

popF<-list()

for(i in 1:1000){

m<-matrix(sample(c(-1,0,1),20,replace=T),ncol=2)

f<-matrix(sample(c(-1,0,1),20,replace=T),ncol=2)

#FOR A 10-GENES MODEL, USE 40 INSTEAD OF 20 IN THE TWO LINES ABOVE

popM[[i]]<-m

popF[[i]]<-f

}

#DEFINING SIZE AND PREFERENCE

library(plyr)

tamanhoM<-lapply(popM, "[", 1:5, 1:2)

tamanhoF<-lapply(popF, "[", 1:5, 1:2)

preferenciaM<-lapply(popM, "[", 6:10, 1:2)

preferenciaF<-lapply(popF, "[", 6:10, 1:2)

#FOR A 10-GENES MODEL, USE 1:10 AND 11:20 IN THE TWO LINES ABOVE

#CHECKING NORMALITY OF INITIAL PHENOTYPES

#tiff("F0-parental.tiff",width = 8, height = 8, units = 'in', res = 300)

par(mfrow=c(2,2))

hist(rnorm(1000,0,1),ylab="Frequency",xlab="Values",main="")

hist(unlist(lapply(tamanhoM,sum)),ylab="",xlab="Male size",main="")

hist(unlist(lapply(tamanhoF,sum)),ylab="Frequency",xlab="Female size",main="")

hist(unlist(lapply(preferenciaF,sum)),ylab="",xlab="Female preference",main="")

#dev.off()

#FOR OVERLAPPING GENERATIONS, DEFINE:

popYF<-NULL

popYM<-NULL

a=numeric(0)

b=numeric(0)

media=NULL

disp=NULL

MEDIA=NULL

DISP=NULL

TAMANHOM=NULL #AND

TAMANHOF=NULL #RIGHT NOW

for(S in 1:5){ #RUNNING THE MODEL FOR 5 GENERATIONS

#FOR OVERLAPPING GENERATIONS, DEFINE:

popYF<-NULL # AND

popYM<-NULL #RIGHT NOW

#DEFINING SIZE AND PREFERENCE

tamanhoM<-lapply(popM, "[", 1:5, 1:2)

tamanhoF<-lapply(popF, "[", 1:5, 1:2)

preferenciaM<-lapply(popM, "[", 6:10, 1:2)

preferenciaF<-lapply(popF, "[", 6:10, 1:2)

#FOR A 10-GENES MODEL, USE 1:10 AND 11:20 IN THE TWO LINES ABOVE

#CREATING FEMALES’ PREFERENCE INTERCEPTS AND SLOPES

sigma<-matrix(c(19.408,10.4,10.4,5.573),2,2)

library(MASS)

param<-mvrnorm(n=1050,mu=c(0.9587,0.5262),sigma)

a=numeric(0)

a[i]<-sample(sort(int)[1:25],1)

for(i in 1:length(popF)){

if(sum(preferenciaF[[i]][1:5,1:2])==-10){

a[i]<-sample(sort(int)[1:50],1)

}else if(sum(preferenciaF[[i]][1:5,1:2])==-9){

a[i]<-sample(sort(int)[51:100],1)

}else if(sum(preferenciaF[[i]][1:5,1:2])==-8){

a[i]<-sample(sort(int)[101:150],1)

}else if(sum(preferenciaF[[i]][1:5,1:2])==-7){

a[i]<-sample(sort(int)[151:200],1)

}else if(sum(preferenciaF[[i]][1:5,1:2])==-6){

a[i]<-sample(sort(int)[201:250],1)

}else if(sum(preferenciaF[[i]][1:5,1:2])==-5){

a[i]<-sample(sort(int)[251:300],1)

}else if(sum(preferenciaF[[i]][1:5,1:2])==-4){

a[i]<-sample(sort(int)[301:350],1)

}else if(sum(preferenciaF[[i]][1:5,1:2])==-3){

a[i]<-sample(sort(int)[351:400],1)

}else if(sum(preferenciaF[[i]][1:5,1:2])==-2){

a[i]<-sample(sort(int)[401:450],1)

}else if(sum(preferenciaF[[i]][1:5,1:2])==-1){

a[i]<-sample(sort(int)[451:500],1)

}else if(sum(preferenciaF[[i]][1:5,1:2])==0){

a[i]<-sample(sort(int)[501:550],1)

}else if(sum(preferenciaF[[i]][1:5,1:2])==1){

a[i]<-sample(sort(int)[551:600],1)

}else if(sum(preferenciaF[[i]][1:5,1:2])==2){

a[i]<-sample(sort(int)[601:650],1)

}else if(sum(preferenciaF[[i]][1:5,1:2])==3){

a[i]<-sample(sort(int)[651:700],1)

}else if(sum(preferenciaF[[i]][1:5,1:2])==4){

a[i]<-sample(sort(int)[701:750],1)

}else if(sum(preferenciaF[[i]][1:5,1:2])==5){

a[i]<-sample(sort(int)[751:800],1)

}else if(sum(preferenciaF[[i]][1:5,1:2])==6){

a[i]<-sample(sort(int)[801:850],1)

}else if(sum(preferenciaF[[i]][1:5,1:2])==7){

a[i]<-sample(sort(int)[851:900],1)

}else if(sum(preferenciaF[[i]][1:5,1:2])==8){

a[i]<-sample(sort(int)[901:950],1)

}else if(sum(preferenciaF[[i]][1:5,1:2])==9){

a[i]<-sample(sort(int)[951:1000],1)

}else{

a[i]<-sample(sort(int)[1001:1050],1)

}

}

b=numeric(0)

for(i in 1:length(popF)){

if(sum(preferenciaF[[i]][1:5,1:2])==-10){

b[i]<-sample(sort(slop)[1:50],1)

}else if(sum(preferenciaF[[i]][1:5,1:2])==-9){

b[i]<-sample(sort(slop)[51:100],1)

}else if(sum(preferenciaF[[i]][1:5,1:2])==-8){

b[i]<-sample(sort(slop)[101:150],1)

}else if(sum(preferenciaF[[i]][1:5,1:2])==-7){

b[i]<-sample(sort(slop)[151:200],1)

}else if(sum(preferenciaF[[i]][1:5,1:2])==-6){

b[i]<-sample(sort(slop)[201:250],1)

}else if(sum(preferenciaF[[i]][1:5,1:2])==-5){

b[i]<-sample(sort(slop)[251:300],1)

}else if(sum(preferenciaF[[i]][1:5,1:2])==-4){

b[i]<-sample(sort(slop)[301:350],1)

}else if(sum(preferenciaF[[i]][1:5,1:2])==-3){

b[i]<-sample(sort(slop)[351:400],1)

}else if(sum(preferenciaF[[i]][1:5,1:2])==-2){

b[i]<-sample(sort(slop)[401:450],1)

}else if(sum(preferenciaF[[i]][1:5,1:2])==-1){

b[i]<-sample(sort(slop)[451:500],1)

}else if(sum(preferenciaF[[i]][1:5,1:2])==0){

b[i]<-sample(sort(slop)[501:550],1)

}else if(sum(preferenciaF[[i]][1:5,1:2])==1){

b[i]<-sample(sort(slop)[551:600],1)

}else if(sum(preferenciaF[[i]][1:5,1:2])==2){

b[i]<-sample(sort(slop)[601:650],1)

}else if(sum(preferenciaF[[i]][1:5,1:2])==3){

b[i]<-sample(sort(slop)[651:700],1)

}else if(sum(preferenciaF[[i]][1:5,1:2])==4){

b[i]<-sample(sort(slop)[701:750],1)

}else if(sum(preferenciaF[[i]][1:5,1:2])==5){

b[i]<-sample(sort(slop)[751:800],1)

}else if(sum(preferenciaF[[i]][1:5,1:2])==6){

b[i]<-sample(sort(slop)[801:850],1)

}else if(sum(preferenciaF[[i]][1:5,1:2])==7){

b[i]<-sample(sort(slop)[851:900],1)

}else if(sum(preferenciaF[[i]][1:5,1:2])==8){

b[i]<-sample(sort(slop)[901:950],1)

}else if(sum(preferenciaF[[i]][1:5,1:2])==9){

b[i]<-sample(sort(slop)[951:1000],1)

}else{

b[i]<-sample(sort(slop)[1001:1050],1)

}

}

#FOR OVELAPPING GENERATIONS, CREATE INTERCEPTS AND SLOPES AS:

A=NULL

B=NULL

if(S==1){

sigma<-matrix(c(19.408,10.4,10.4,5.573),2,2)

library(MASS)

param<-mvrnorm(n=1050,mu=c(0.9587,0.5262),sigma)

for(i in 1:length(popF)){

if(sum(preferenciaF[[i]][1:5,1:2])==-10){

a[i]<-sample(sort(int)[1:50],1)

}else if(sum(preferenciaF[[i]][1:5,1:2])==-9){

a[i]<-sample(sort(int)[51:100],1)

}else if(sum(preferenciaF[[i]][1:5,1:2])==-8){

a[i]<-sample(sort(int)[101:150],1)

}else if(sum(preferenciaF[[i]][1:5,1:2])==-7){

a[i]<-sample(sort(int)[151:200],1)

}else if(sum(preferenciaF[[i]][1:5,1:2])==-6){

a[i]<-sample(sort(int)[201:250],1)

}else if(sum(preferenciaF[[i]][1:5,1:2])==-5){

a[i]<-sample(sort(int)[251:300],1)

}else if(sum(preferenciaF[[i]][1:5,1:2])==-4){

a[i]<-sample(sort(int)[301:350],1)

}else if(sum(preferenciaF[[i]][1:5,1:2])==-3){

a[i]<-sample(sort(int)[351:400],1)

}else if(sum(preferenciaF[[i]][1:5,1:2])==-2){

a[i]<-sample(sort(int)[401:450],1)

}else if(sum(preferenciaF[[i]][1:5,1:2])==-1){

a[i]<-sample(sort(int)[451:500],1)

}else if(sum(preferenciaF[[i]][1:5,1:2])==0){

a[i]<-sample(sort(int)[501:550],1)

}else if(sum(preferenciaF[[i]][1:5,1:2])==1){

a[i]<-sample(sort(int)[551:600],1)

}else if(sum(preferenciaF[[i]][1:5,1:2])==2){

a[i]<-sample(sort(int)[601:650],1)

}else if(sum(preferenciaF[[i]][1:5,1:2])==3){

a[i]<-sample(sort(int)[651:700],1)

}else if(sum(preferenciaF[[i]][1:5,1:2])==4){

a[i]<-sample(sort(int)[701:750],1)

}else if(sum(preferenciaF[[i]][1:5,1:2])==5){

a[i]<-sample(sort(int)[751:800],1)

}else if(sum(preferenciaF[[i]][1:5,1:2])==6){

a[i]<-sample(sort(int)[801:850],1)

}else if(sum(preferenciaF[[i]][1:5,1:2])==7){

a[i]<-sample(sort(int)[851:900],1)

}else if(sum(preferenciaF[[i]][1:5,1:2])==8){

a[i]<-sample(sort(int)[901:950],1)

}else if(sum(preferenciaF[[i]][1:5,1:2])==9){

a[i]<-sample(sort(int)[951:1000],1)

}else{

a[i]<-sample(sort(int)[1001:1050],1)

}

}

}else{

sigma<-matrix(c(6.224526,3.33563,3.33563,1.787515),2,2)

library(MASS)

param<-mvrnorm(n=1050,mu=c(0.9499329,0.5215195),sigma)

for(i in 1:length(popF %in% popYF[[S-1]])){

if(sum(preferenciaF[[i]][1:5,1:2])==-10){

A[i]<-sample(sort(int)[1:50],1)

}else if(sum(preferenciaF[[i]][1:5,1:2])==-9){

A[i]<-sample(sort(int)[51:100],1)

}else if(sum(preferenciaF[[i]][1:5,1:2])==-8){

A[i]<-sample(sort(int)[101:150],1)

}else if(sum(preferenciaF[[i]][1:5,1:2])==-7){

A[i]<-sample(sort(int)[151:200],1)

}else if(sum(preferenciaF[[i]][1:5,1:2])==-6){

A[i]<-sample(sort(int)[201:250],1)

}else if(sum(preferenciaF[[i]][1:5,1:2])==-5){

A[i]<-sample(sort(int)[251:300],1)

}else if(sum(preferenciaF[[i]][1:5,1:2])==-4){

A[i]<-sample(sort(int)[301:350],1)

}else if(sum(preferenciaF[[i]][1:5,1:2])==-3){

A[i]<-sample(sort(int)[351:400],1)

}else if(sum(preferenciaF[[i]][1:5,1:2])==-2){

A[i]<-sample(sort(int)[401:450],1)

}else if(sum(preferenciaF[[i]][1:5,1:2])==-1){

A[i]<-sample(sort(int)[451:500],1)

}else if(sum(preferenciaF[[i]][1:5,1:2])==0){

A[i]<-sample(sort(int)[501:550],1)

}else if(sum(preferenciaF[[i]][1:5,1:2])==1){

A[i]<-sample(sort(int)[551:600],1)

}else if(sum(preferenciaF[[i]][1:5,1:2])==2){

A[i]<-sample(sort(int)[601:650],1)

}else if(sum(preferenciaF[[i]][1:5,1:2])==3){

A[i]<-sample(sort(int)[651:700],1)

}else if(sum(preferenciaF[[i]][1:5,1:2])==4){

A[i]<-sample(sort(int)[701:750],1)

}else if(sum(preferenciaF[[i]][1:5,1:2])==5){

A[i]<-sample(sort(int)[751:800],1)

}else if(sum(preferenciaF[[i]][1:5,1:2])==6){

A[i]<-sample(sort(int)[801:850],1)

}else if(sum(preferenciaF[[i]][1:5,1:2])==7){

A[i]<-sample(sort(int)[851:900],1)

}else if(sum(preferenciaF[[i]][1:5,1:2])==8){

A[i]<-sample(sort(int)[901:950],1)

}else if(sum(preferenciaF[[i]][1:5,1:2])==9){

A[i]<-sample(sort(int)[951:1000],1)

}else{

A[i]<-sample(sort(int)[1001:1050],1)

}

}

}

a<-c(A,a)

if(S==1){

for(i in 1:length(popF)){

if(sum(preferenciaF[[i]][1:5,1:2])==-10){

b[i]<-sample(sort(slop)[1:50],1)

}else if(sum(preferenciaF[[i]][1:5,1:2])==-9){

b[i]<-sample(sort(slop)[51:100],1)

}else if(sum(preferenciaF[[i]][1:5,1:2])==-8){

b[i]<-sample(sort(slop)[101:150],1)

}else if(sum(preferenciaF[[i]][1:5,1:2])==-7){

b[i]<-sample(sort(slop)[151:200],1)

}else if(sum(preferenciaF[[i]][1:5,1:2])==-6){

b[i]<-sample(sort(slop)[201:250],1)

}else if(sum(preferenciaF[[i]][1:5,1:2])==-5){

b[i]<-sample(sort(slop)[251:300],1)

}else if(sum(preferenciaF[[i]][1:5,1:2])==-4){

b[i]<-sample(sort(slop)[301:350],1)

}else if(sum(preferenciaF[[i]][1:5,1:2])==-3){

b[i]<-sample(sort(slop)[351:400],1)

}else if(sum(preferenciaF[[i]][1:5,1:2])==-2){

b[i]<-sample(sort(slop)[401:450],1)

}else if(sum(preferenciaF[[i]][1:5,1:2])==-1){

b[i]<-sample(sort(slop)[451:500],1)

}else if(sum(preferenciaF[[i]][1:5,1:2])==0){

b[i]<-sample(sort(slop)[501:550],1)

}else if(sum(preferenciaF[[i]][1:5,1:2])==1){

b[i]<-sample(sort(slop)[551:600],1)

}else if(sum(preferenciaF[[i]][1:5,1:2])==2){

b[i]<-sample(sort(slop)[601:650],1)

}else if(sum(preferenciaF[[i]][1:5,1:2])==3){

b[i]<-sample(sort(slop)[651:700],1)

}else if(sum(preferenciaF[[i]][1:5,1:2])==4){

b[i]<-sample(sort(slop)[701:750],1)

}else if(sum(preferenciaF[[i]][1:5,1:2])==5){

b[i]<-sample(sort(slop)[751:800],1)

}else if(sum(preferenciaF[[i]][1:5,1:2])==6){

b[i]<-sample(sort(slop)[801:850],1)

}else if(sum(preferenciaF[[i]][1:5,1:2])==7){

b[i]<-sample(sort(slop)[851:900],1)

}else if(sum(preferenciaF[[i]][1:5,1:2])==8){

b[i]<-sample(sort(slop)[901:950],1)

}else if(sum(preferenciaF[[i]][1:5,1:2])==9){

b[i]<-sample(sort(slop)[951:1000],1)

}else{

b[i]<-sample(sort(slop)[1001:1050],1)

}

}

}else{

for(i in 1:length(popF %in% popYF[[S-1]])){

if(sum(preferenciaF[[i]][1:5,1:2])==-10){

B[i]<-sample(sort(slop)[1:50],1)

}else if(sum(preferenciaF[[i]][1:5,1:2])==-9){

B[i]<-sample(sort(slop)[51:100],1)

}else if(sum(preferenciaF[[i]][1:5,1:2])==-8){

B[i]<-sample(sort(slop)[101:150],1)

}else if(sum(preferenciaF[[i]][1:5,1:2])==-7){

B[i]<-sample(sort(slop)[151:200],1)

}else if(sum(preferenciaF[[i]][1:5,1:2])==-6){

B[i]<-sample(sort(slop)[201:250],1)

}else if(sum(preferenciaF[[i]][1:5,1:2])==-5){

B[i]<-sample(sort(slop)[251:300],1)

}else if(sum(preferenciaF[[i]][1:5,1:2])==-4){

B[i]<-sample(sort(slop)[301:350],1)

}else if(sum(preferenciaF[[i]][1:5,1:2])==-3){

B[i]<-sample(sort(slop)[351:400],1)

}else if(sum(preferenciaF[[i]][1:5,1:2])==-2){

B[i]<-sample(sort(slop)[401:450],1)

}else if(sum(preferenciaF[[i]][1:5,1:2])==-1){

B[i]<-sample(sort(slop)[451:500],1)

}else if(sum(preferenciaF[[i]][1:5,1:2])==0){

B[i]<-sample(sort(slop)[501:550],1)

}else if(sum(preferenciaF[[i]][1:5,1:2])==1){

B[i]<-sample(sort(slop)[551:600],1)

}else if(sum(preferenciaF[[i]][1:5,1:2])==2){

B[i]<-sample(sort(slop)[601:650],1)

}else if(sum(preferenciaF[[i]][1:5,1:2])==3){

B[i]<-sample(sort(slop)[651:700],1)

}else if(sum(preferenciaF[[i]][1:5,1:2])==4){

B[i]<-sample(sort(slop)[701:750],1)

}else if(sum(preferenciaF[[i]][1:5,1:2])==5){

B[i]<-sample(sort(slop)[751:800],1)

}else if(sum(preferenciaF[[i]][1:5,1:2])==6){

B[i]<-sample(sort(slop)[801:850],1)

}else if(sum(preferenciaF[[i]][1:5,1:2])==7){

B[i]<-sample(sort(slop)[851:900],1)

}else if(sum(preferenciaF[[i]][1:5,1:2])==8){

B[i]<-sample(sort(slop)[901:950],1)

}else if(sum(preferenciaF[[i]][1:5,1:2])==9){

B[i]<-sample(sort(slop)[951:1000],1)

}else{

B[i]<-sample(sort(slop)[1001:1050],1)

}

}

}

b<-c(B,b)

#DEFINING ANIMALS’S SIZES

tamanhom=NULL

for (i in 1:length(popM)){

tamanhom[i]=

sum(tamanhoM[[i]])

#FOR ENVIRONMENTAL EFFECT ON SIZE, USE #sum(tamanhoM[[i]])+rnorm(1,0,1.5) IN THE 5-GENES MODEL AND #sum(tamanhoM[[i]])+rnorm(1,0,2.16) IN THE 10-GENES MODEL

}

zm<-(tamanhom-mean(tamanhom))/(sd(tamanhom))

tamanhof=NULL

for (i in 1:length(popF)){

tamanhof[i]=

sum(tamanhoF[[i]])

#FOR ENVIRONMENTAL EFFECT ON SIZE, USE #sum(tamanhoM[[i]])+rnorm(1,0,1.5) IN THE 5-GENES MODEL AND #sum(tamanhoM[[i]])+rnorm(1,0,2.16) IN THE 10-GENES MODEL

}

zf<-(tamanhof-mean(tamanhof))/(sd(tamanhof))

#CREATING MALES’ MEANS AND SHAPES FOR PREDICTING NUMBER OF YOUNG

media<-NULL

disp<-NULL

for (i in 1:length(popM)){

media[i]=exp(3.36+0.42*zm[i])+rnorm(1,0,13.15)

if(media[i]<0){

media[i]<-0

}

disp[i]=sample(shape,1)

}

#FOR OVERLAPPING GENERATIONS, CREATE MALES’ MEANS AND SHAPES AS:

if(S==1){

for (i in 1:length(popM)){

media[i]=exp(3.36+0.42*zm[i])+rnorm(1,0,13.15)

if(media[i]<0){

media[i]<-0

}

disp[i]=sample(shape,1)

}

}else{

for (i in 1:length(popM %in% popYM[[S-1]])){

MEDIA[i]=exp(3.36+0.42*zm[i])+rnorm(1,0,13.15)

if(MEDIA[i]<0){

MEDIA[i]<-0

}

DISP[i]=sample(shape,1)

}

}

media<-c(MEDIA,media)

disp<-c(DISP,disp)

#PRODUCING YOUNG

#shape NEEDS TO BE SPECIFIED BEFOREHAND

y<-numeric(0)

popYM<-NULL #ONLY IF THERE IS NO OVERLAPPING GENERATIONS

popYF<-NULL #ONLY IF THERE IS NO OVERLAPPING GENERATIONS

young<-matrix(nrow=length(popF),ncol=length(popM))

lay<-matrix(nrow=length(popF),ncol=length(popM))

copulate<-matrix(nrow=length(popF),ncol=length(popM))

for(i in 1:length(popF)){

for(j in 1:length(popM)){

popy<-NULL

popf<-NULL

popm<-NULL

copulate[i,j]<-rbinom(1,1,(exp(a[i]-2.9*zf[i]+b[i]*zm[j])/(1+(exp(a[i]-2.9*zf[i]+b[i]*zm[j])))))

if(copulate[i,j]==1){

lay[i,j]<-rbinom(1,1,0.695)

}else{

lay[i,j]<-0

}

if(lay[i,j]==1){

young[i,j]<-rnbinom(n=1,s=disp[j],mu=media[j])

}else {

young[i,j]<-0

}

if(young[i,j]>0){

for(k in 1:young[i,j]) {

popy[[k]]<-cbind(c(

popF[[i]][1,sample(1:2,1)],

popF[[i]][2,sample(1:2,1)],

popF[[i]][3,sample(1:2,1)],

popF[[i]][4,sample(1:2,1)],

popF[[i]][5,sample(1:2,1)],

popF[[i]][6,sample(1:2,1)],

popF[[i]][7,sample(1:2,1)],

popF[[i]][8,sample(1:2,1)],

popF[[i]][9,sample(1:2,1)],

popF[[i]][10,sample(1:2,1)]),

c(popM[[j]][1,sample(1:2,1)],

popM[[j]][2,sample(1:2,1)],

popM[[j]][3,sample(1:2,1)],

popM[[j]][4,sample(1:2,1)],

popM[[j]][5,sample(1:2,1)],

popM[[j]][6,sample(1:2,1)],

popM[[j]][7,sample(1:2,1)],

popM[[j]][8,sample(1:2,1)],

popM[[j]][9,sample(1:2,1)],

popM[[j]][10,sample(1:2,1)]))

popy[1:round(length(popy)/2)]->popf

popy[(round(length(popy)/2)+1):length(popy)]->popm

}

}

if(copulate[i,j]==1){

break

}

}

#UPDATING PARENTAL POPULATION

popYF<-c(popYF,popf)

popYM<-c(popYM,popm)

}

popF<-popYF

popM<-popYM

if(length(popF)<10000){

popF<-popF[1:10000]

popF = popF[-which(sapply(popF, is.null))]

}else{

popF<-sample(popF,size=10000)

}

if(length(popM)<10000){

popM<-popM[1:10000]

popM = popM[-which(sapply(popM, is.null))]

}else{

popM<-sample(popM,size=10000)

}

}

#IF THERE IS OVERLAPPING GENERATIONS, UPDATE PARENTAL POPULATION AS:

popYF[[S]]<-c(popYF[[S]],popf)

popYM[[S]]<-c(popYM[[S]],popm)

}

if(S>1){

popM<-c(popYM[[S-1]],popYM[[S]])

popF<-c(popYF[[S-1]],popYF[[S]])

}else{

popM<-c(popM,popYM[[S]])

popF<-c(popF,popYF[[S]])

}

if(length(popM)<10000){

popM<-popM[1:10000]

popM = popM[-which(sapply(popM, is.null))]

}else{

popM<-sample(popM,size=10000)

}

if(length(popF)<10000){

popF<-popF[1:10000]

popF = popF[-which(sapply(popF, is.null))]

}else{

popF<-sample(popF,size=10000)

}

#DEFINING MUTATION RATE

for (i in 1:length(popM)){

for(a in 1:2){

for(g in 1:10){

x=rbinom(1,1,0.01)

if(x==1){

popM[[i]][g,a]<-sample(c(-1,0,1),size=1)

}else{

popM[[i]][g,a]<-popM[[i]][g,a]

}

}

}

}

for (i in 1:length(popF)){

for(a in 1:2){

for(g in 1:10){

x=rbinom(1,1,0.01)

if(x==1){

popF[[i]][g,a]<-sample(c(-1,0,1),size=1)

}else{

popF[[i]][g,a]<-popF[[i]][g,a]

}

}

}

}

}

#tiff("z.tiff",width = 16, height = 10, units = 'in', res = 300)

par(mar=c(10,10,4,2),mfrow=c(1,3))

hist(unlist(lapply(tamanhoM,sum)),main="",xlab="Male size",xlim=c(-10,10),cex.lab=2.5,cex.axis=2.5)

hist(unlist(lapply(tamanhoF,sum)),main="",ylab="",xlab="Female size",xlim=c(-10,10),cex.lab=2.5,cex.axis=2.5)

hist(unlist(lapply(preferenciaF,sum)),main="",ylab="",xlab="Female preference",xlim=c(-10,10),cex.lab=2.5,cex.axis=2.5)

#dev.off()

#THE END

#HOW TO EXTRACT THE MIXED-MODEL PARAMETERS TO INCLUDE IN THE SIMULATIONS

data<-read.table("data.txt",h=T)

attach(data)

#CONVERTING VARIABLES TO Z-SCORES

fz<-(FemaleSize-mean(FemaleSize,na.rm=T))/sd(FemaleSize,na.rm=T)

mz<-(MaleSize-mean(MaleSize,na.rm=T))/sd(MaleSize,na.rm=T)

library(lme4)

mod<-glmer(Success~mz+fz+(mz|FemaleID),family=binomial)

#TO SEE VARIANCES AND MEANS OF ALL PARAMETERS

summary(mod)

#TO SEE THE VARIANCE-COVARIANCE MATRIX OF RANDOM PARAMETERS

print(VarCorr(mod),comp=c('Variance'))
