# Supplementary material for: Divergent preference functions generate directional selection in a jumping spider
Source: Sci Rep. 2023 Dec 21;13:22794. doi: 10.1038/s41598-023-50241-x (PMC10739821; doi:10.1038/s41598-023-50241-x)
Supplement: Supplementary file 2 — Supplementary Figures. [file 41598_2023_50241_MOESM2_ESM.docx]

**Divergent preference functions generate directional selection in a jumping spider**

Behavioral Ecology and Sociobiology

Leonardo Braga Castilho

[leonardobcastilho@gmail.com](mailto:leonardobcastilho@gmail.com)


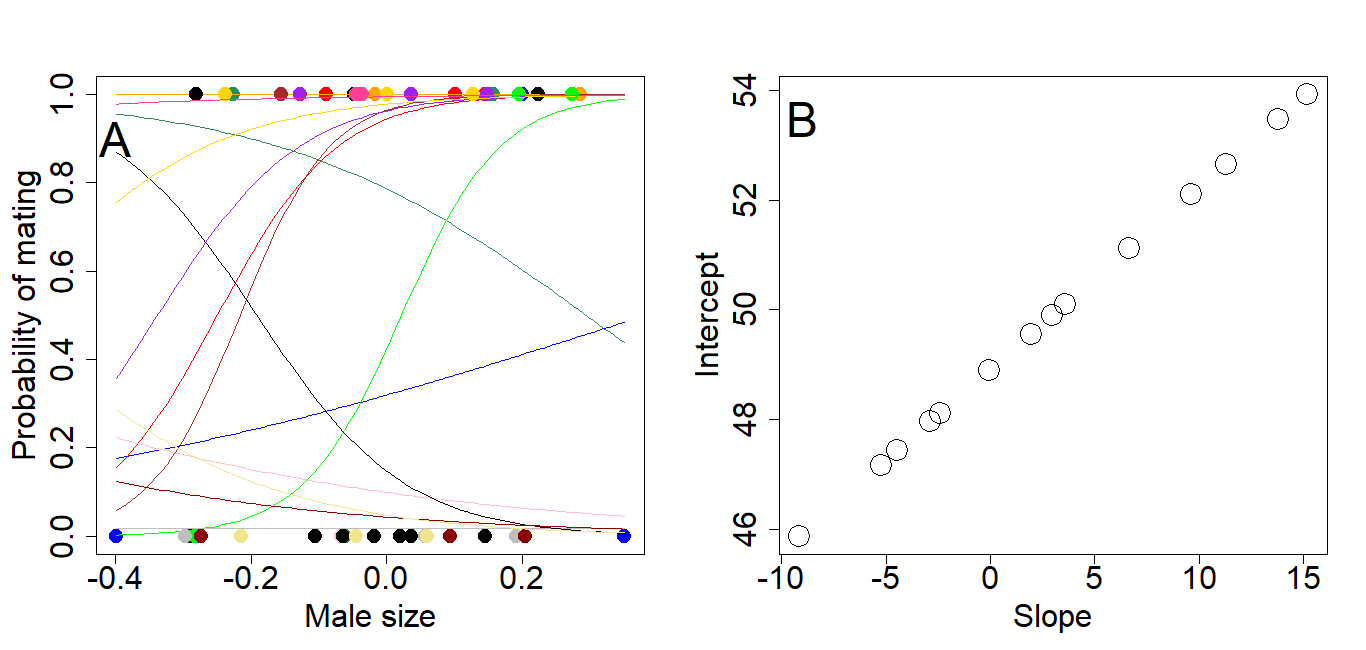


Fig S1 Relationship between probability of a female copulating with a male and male’s size in *Hasarius adansoni*. (A) Each color represents a different female, and male size was centered by its mean. The plot was extracted from a mixed binomial model with female identity as random factor. (B) Correlation between intercept and slope of females seen in A.


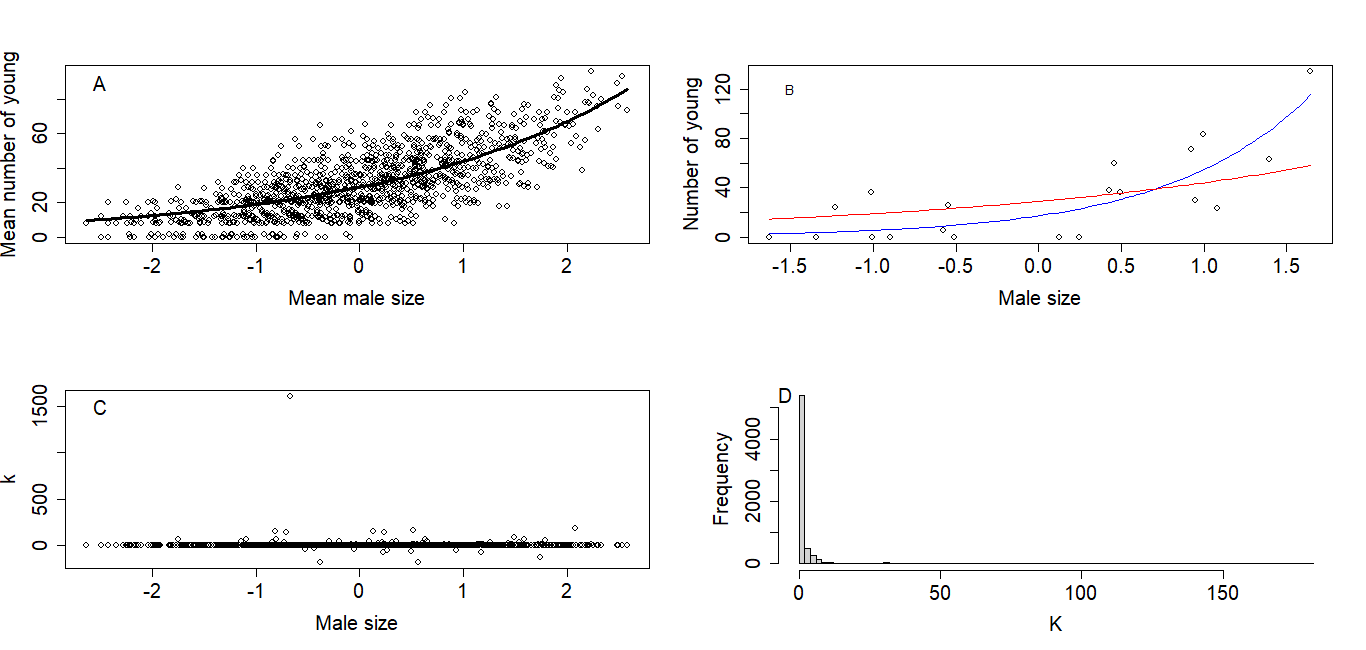


Fig S2 Relationship between *Hasarius adansoni* male body size (standardized to z scores), and the parameters of the negative binomial distribution which approximates the probability distribution of number of young a male sires. To build these graphics, a total of 20 males with known number of young were permuted in groups of 3, making up a total of 6,840 groups. For each group, I extracted mean body size, mean number of young (µ), and the dispersion parameter (k). (A) I plotted mean body size and µ, and the plot shows a positive exponential relationship. (B) When plotting only the original 20 males, a similar trend is shown. The blue line is the exponential line calculated based on the original 20 males. The red line is plotted using the parameters from the permuted groups shown in A. (C) I also plotted mean body size and k, but no clear relationship was found. (D) After removing one outlier and the few negative values, I was able to create a realistic probability distribution of k (called K), which does not depend on male size and is thought to vary randomly among males.


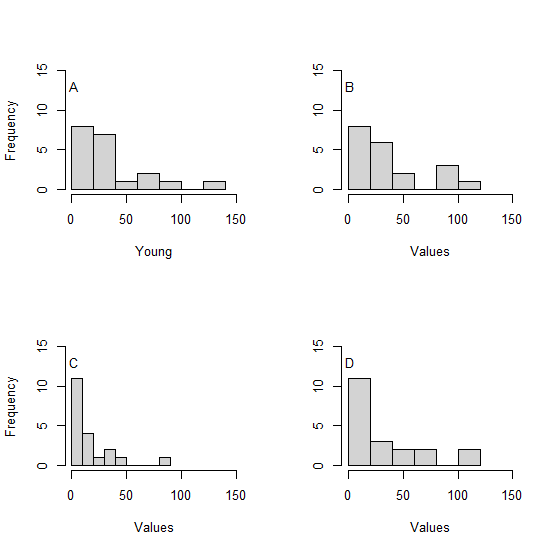


Fig S3 Comparison between number of young produced by *Hasarius adansoni* males and a negative binomial distribution. (A) Real values of number of young produced by males *H. adansoni*. These values had sample size equal to n=20, mean equal to µ=31.45, and a dispersion parameter equal to k=0.789. (B-E) Three histograms of random samples from a negative binomial distribution, with the same n, µ, and k as observed in A. The comparision between the original values of number of young and the negative binomial distribution samples clearly shows that number of young follow such distribution.


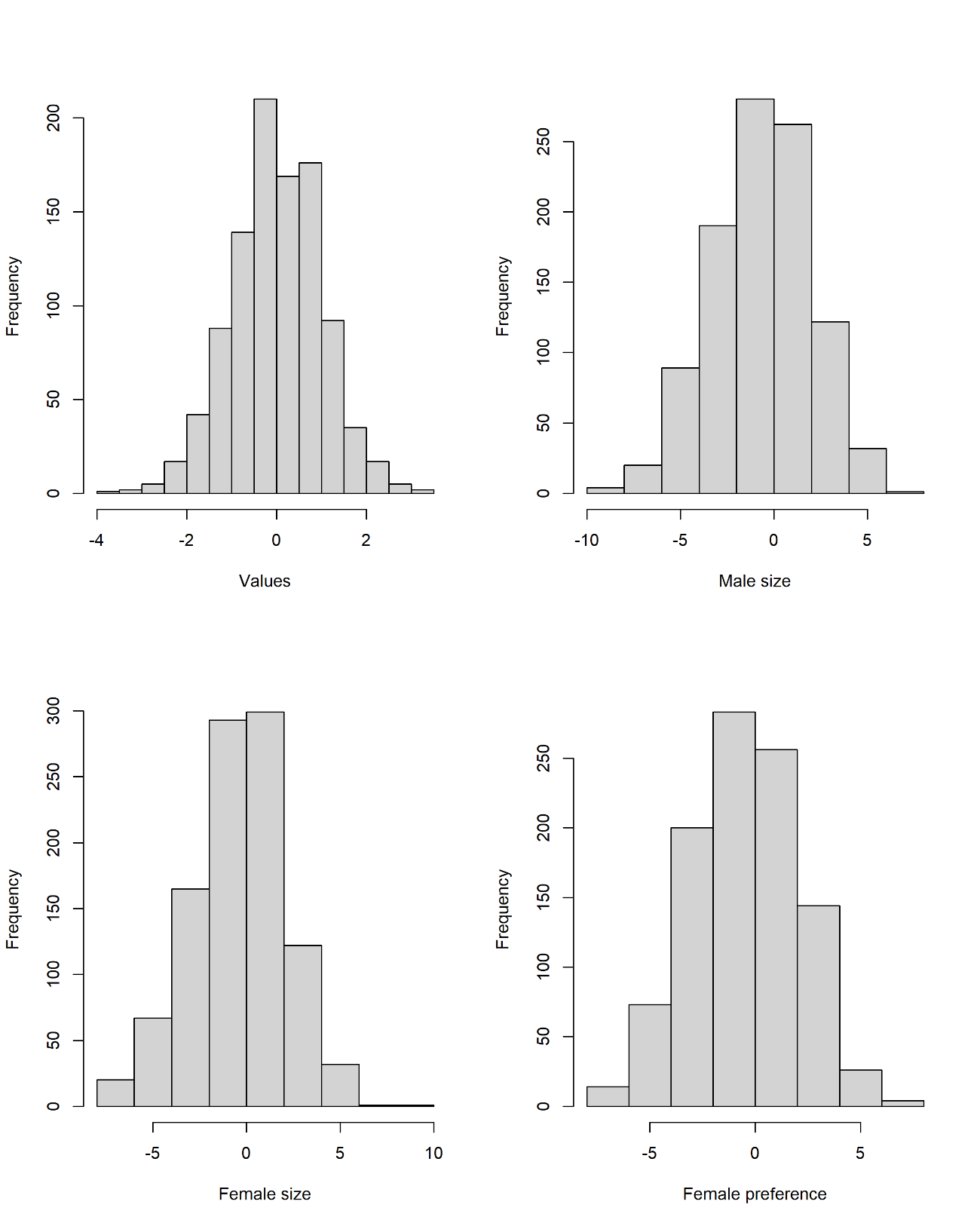


**D**

**C**

**A**

**B**

Fig S4 Initial values simulated for *Hasarius adansoni* phenotypes for the 5-genes+environment model. (A) A random sample from a normal distribution, for comparison. (B) Male size. (C) Female size. (D) Female preference.

**A**


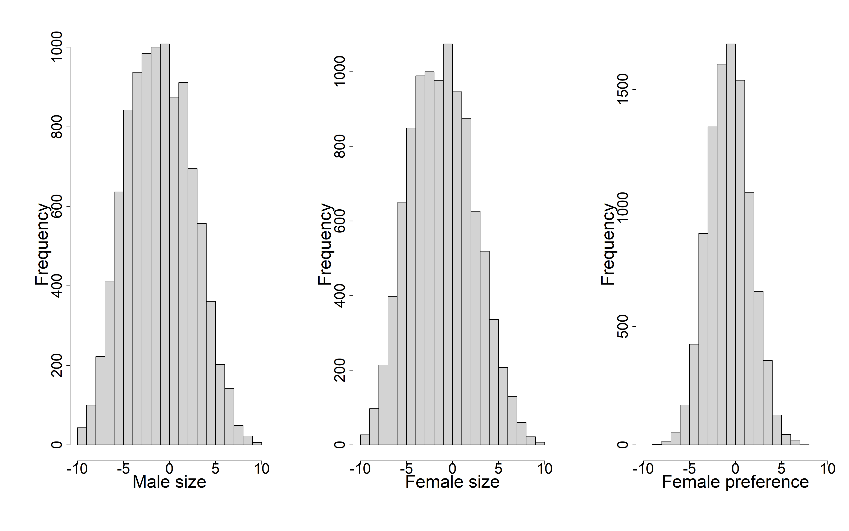

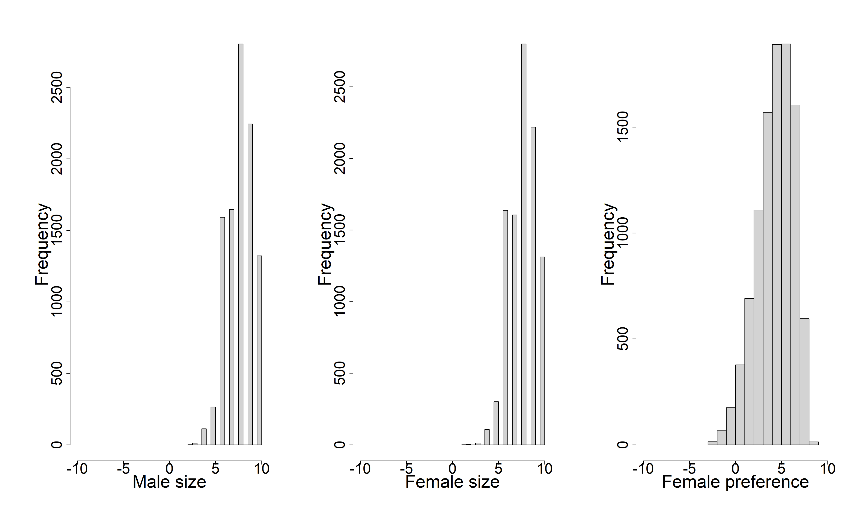

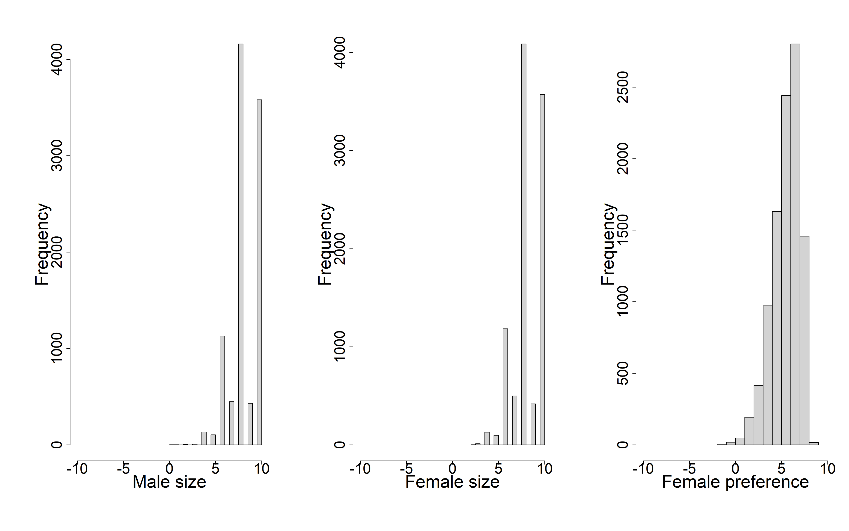

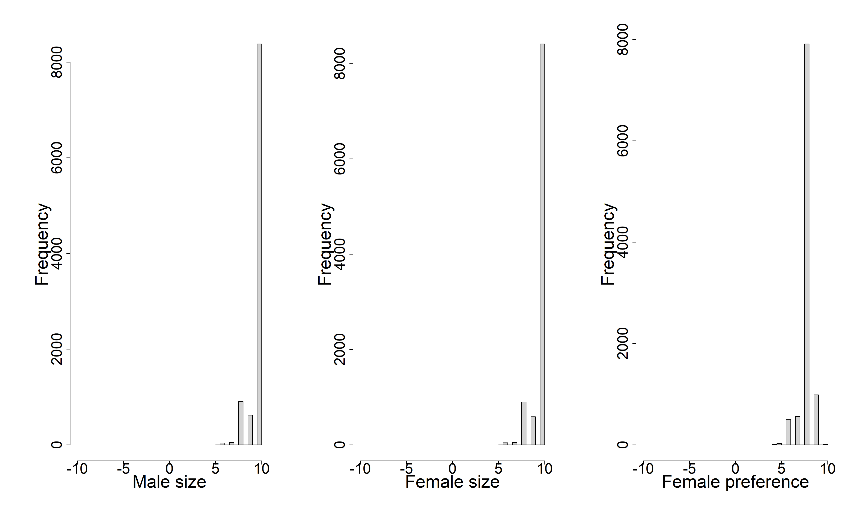


**B**

**C**

**D**

Fig S5 Evolutionary path of sizes and female preferences of *Hasarius adansoni* in the 5-genes+environment model. The figure shows frequency distributions of all phenotypes after (A) 5 generations, (B) 25 generations, (C) 45 generations, and (D) 75 generations. Parental frequency distributions are shown in Fig S4.


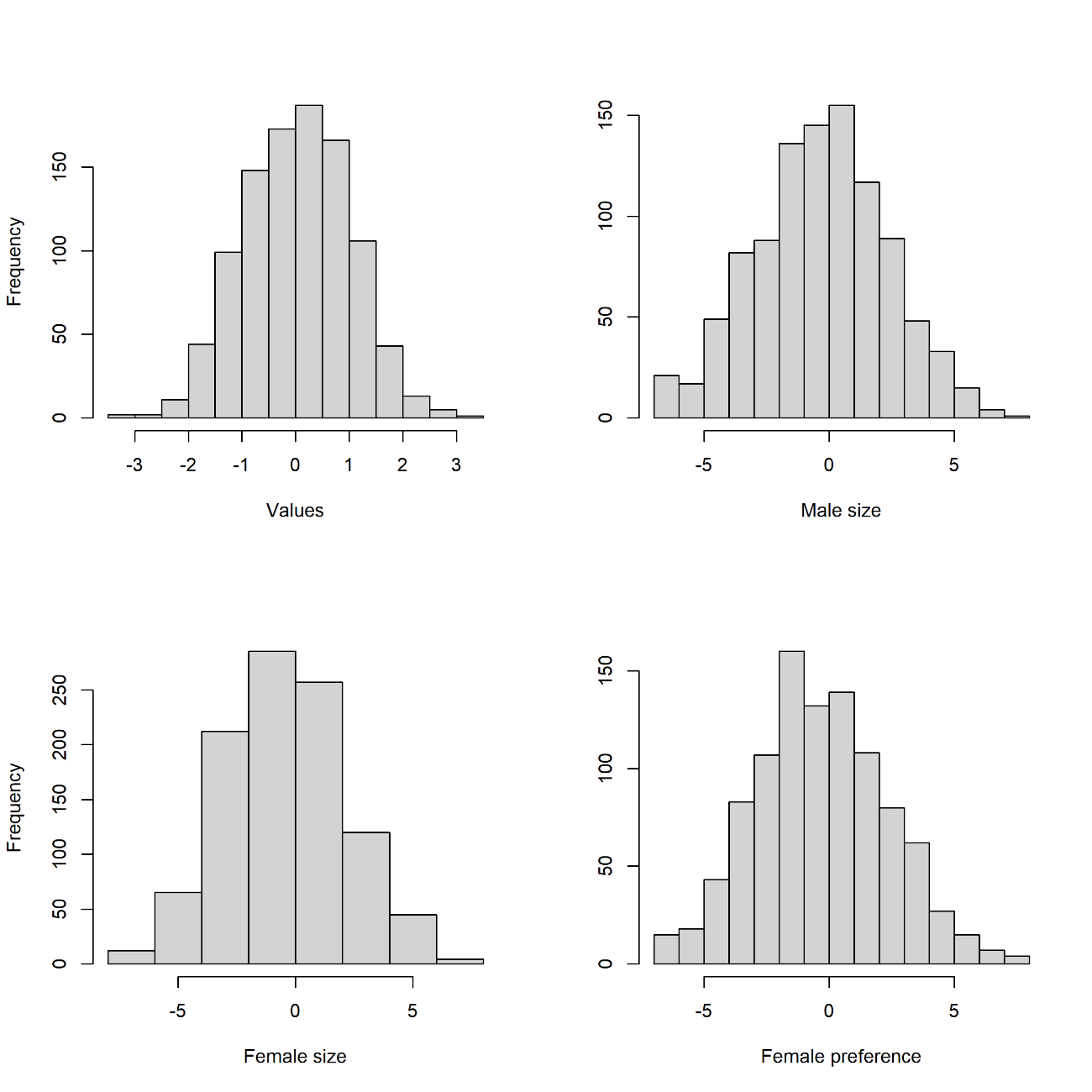


**D**

**C**

**B**

**A**

Fig S6 Initial values simulated for *Hasarius adansoni* phenotypes for the 5-genes+overlap model. (A) A random sample from a normal distribution, for comparison. (B) Male size. (C) Female size. (D) Female preference.

**A**


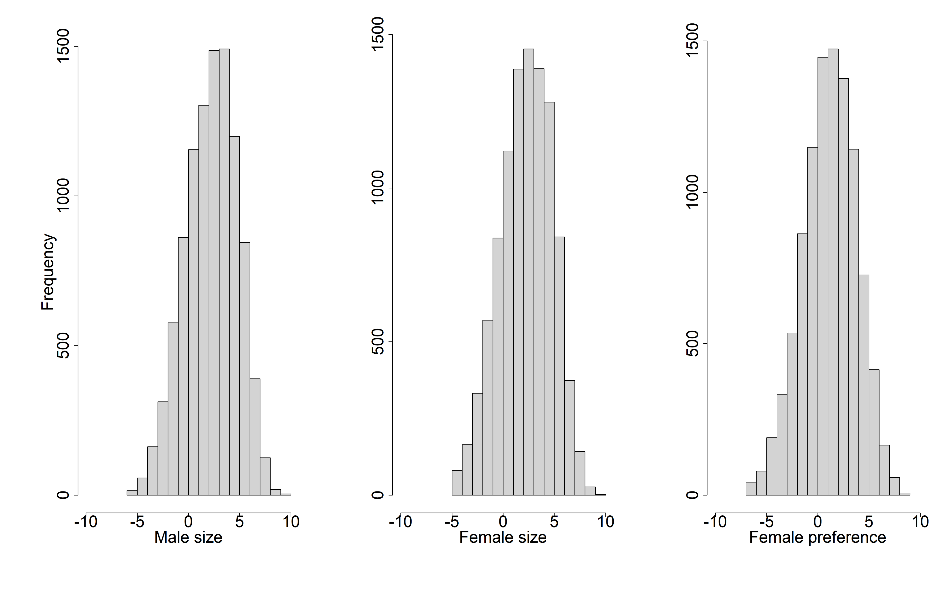

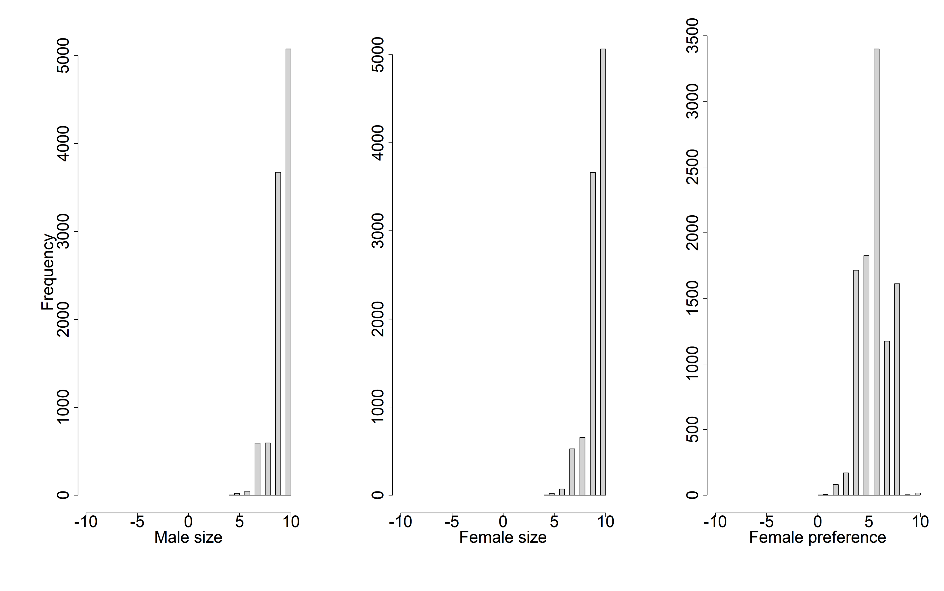

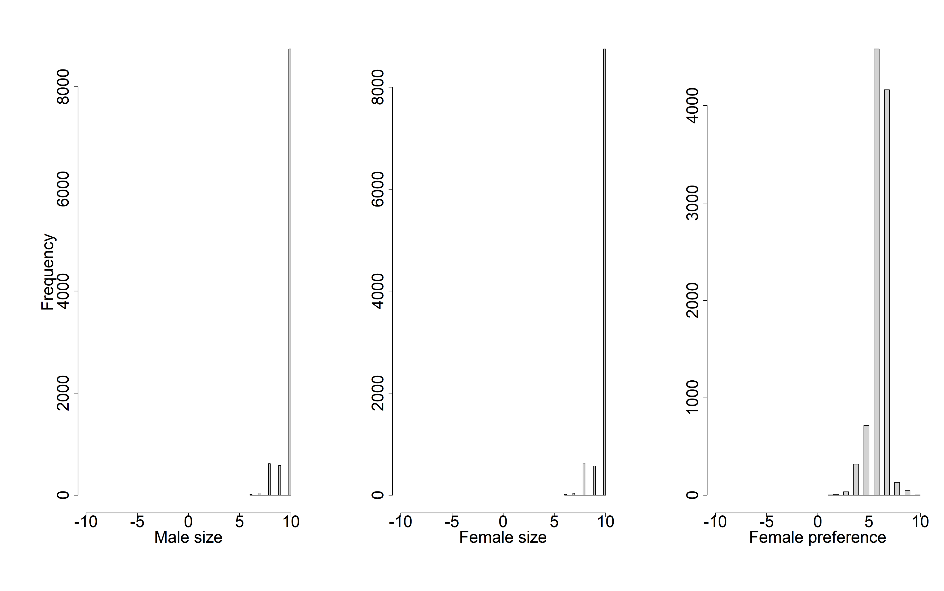

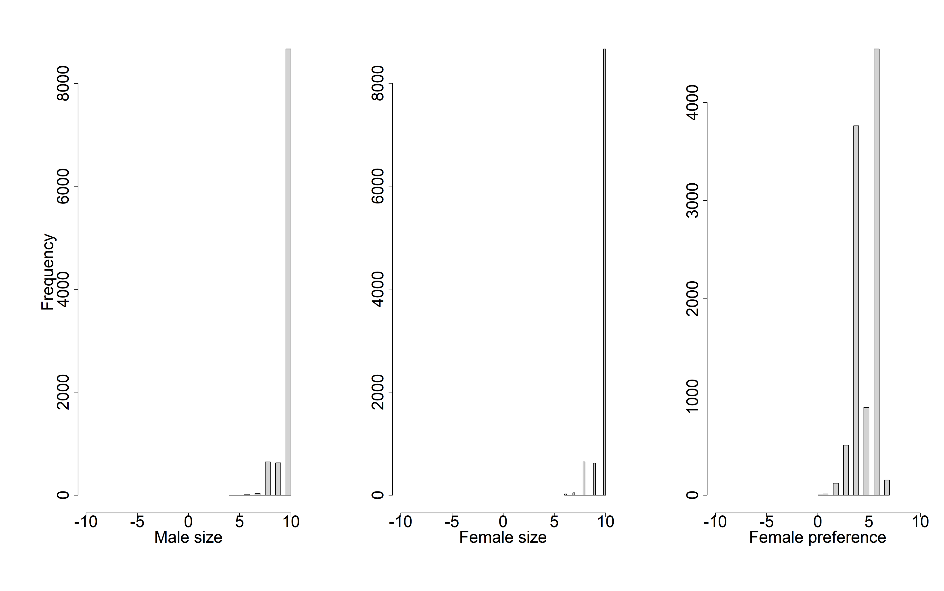


**B**

**C**

**D**

Fig S7 Evolutionary path of sizes and female preferences of *Hasarius adansoni* in the 5-genes+overlap model. The figure shows frequency distributions of all phenotypes after (A) 5 generations, (B) 25 generations, (C) 45 generations, and (D) 75 generations. Parental frequency distributions are shown in Fig S6.


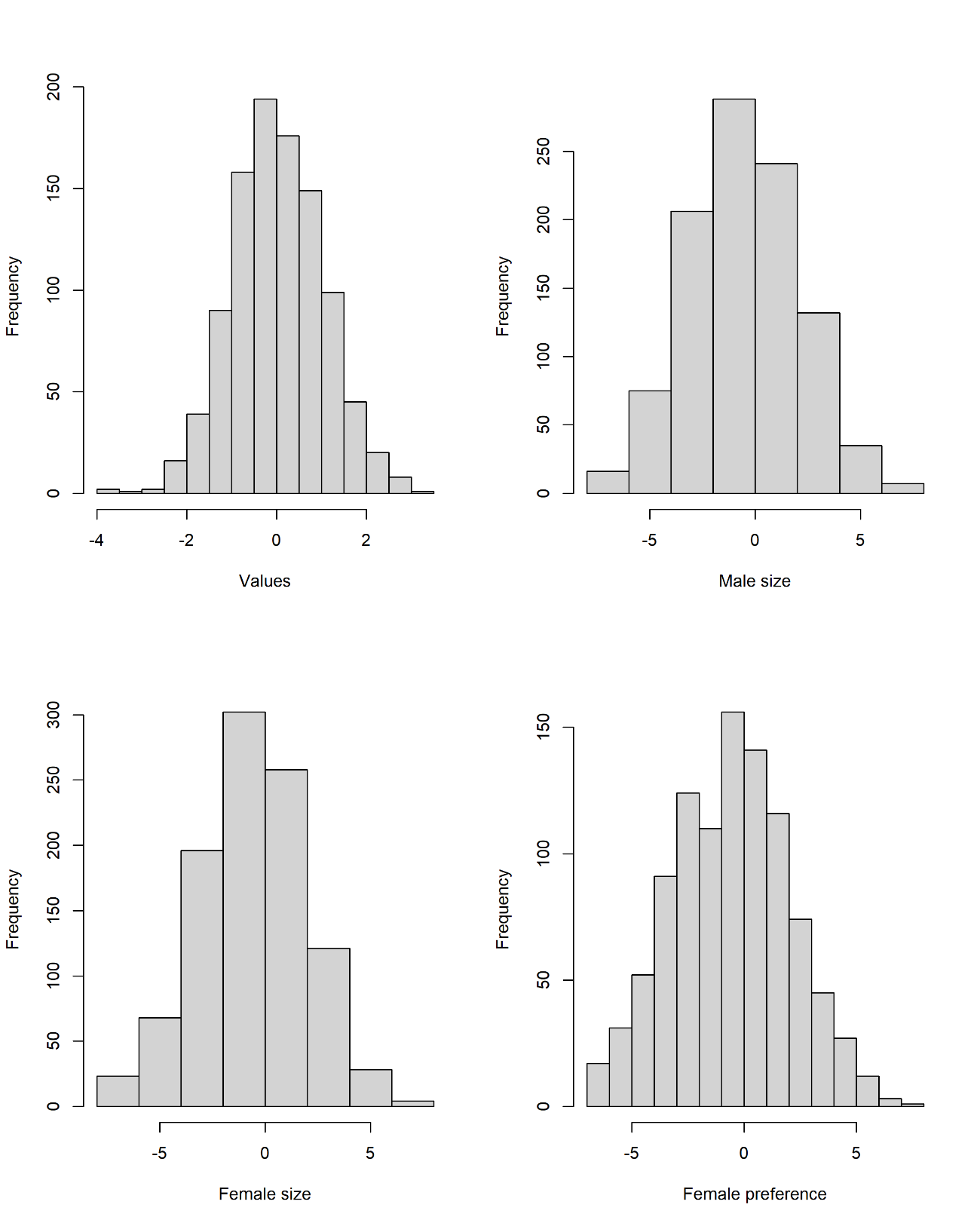


**B**

**A**

**D**

**C**

Fig S8 Initial values simulated for *Hasarius adansoni* phenotypes for the 5-genes+environment+overlap model. (A) A random sample from a normal distribution, for comparison. (B) Male size. (C) Female size. (D) Female preference.


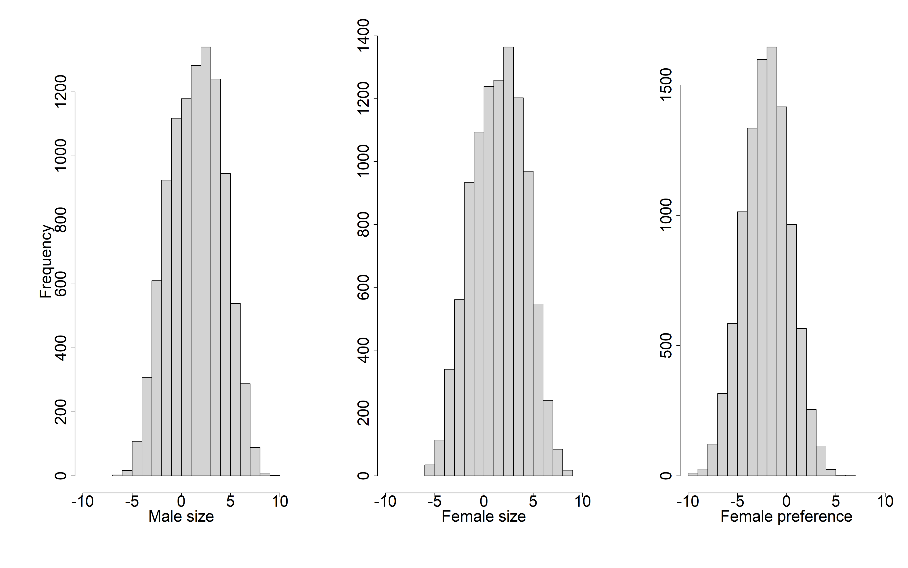

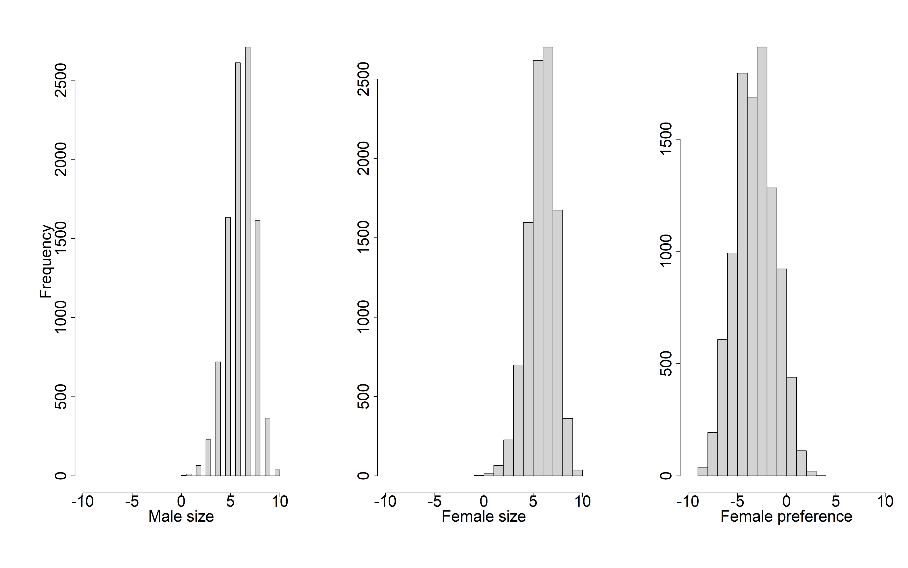

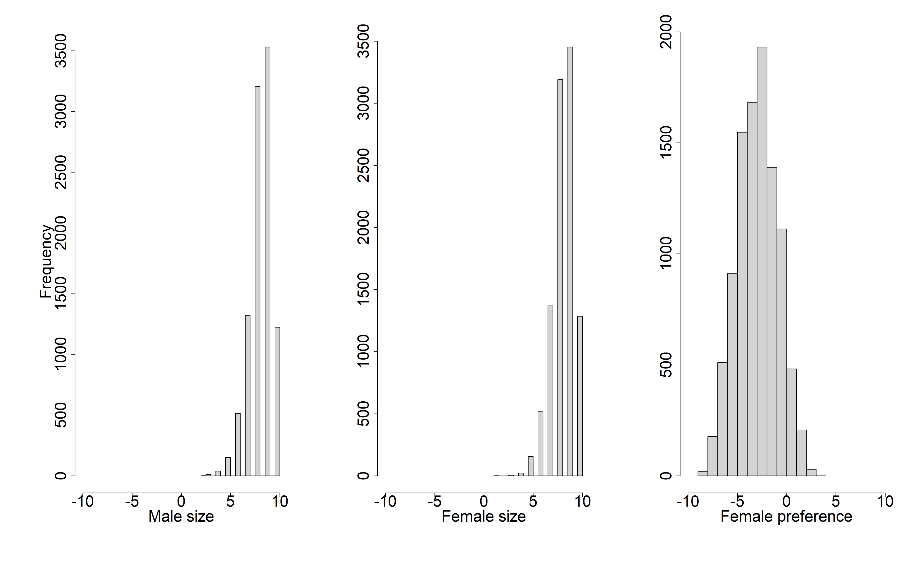

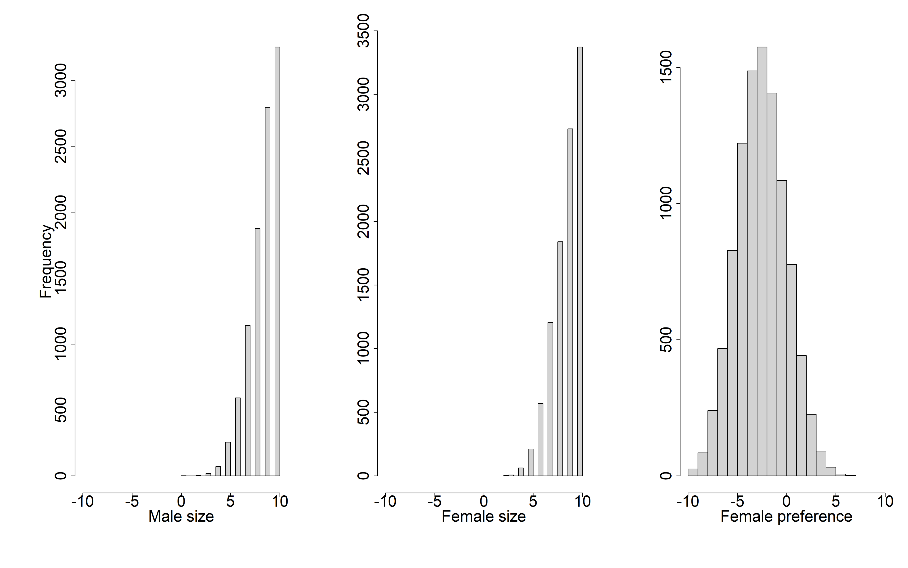


**A**

**B**

**C**

**D**

Fig S9 Evolutionary path of sizes and female preferences of *Hasarius adansoni* in the 5-genes+environment+overlap model. The figure shows frequency distributions of all phenotypes after (A) 5 generations, (B) 25 generations, (C) 45 generations, and (D) 75 generations. Parental frequency distributions are shown in Fig S8.


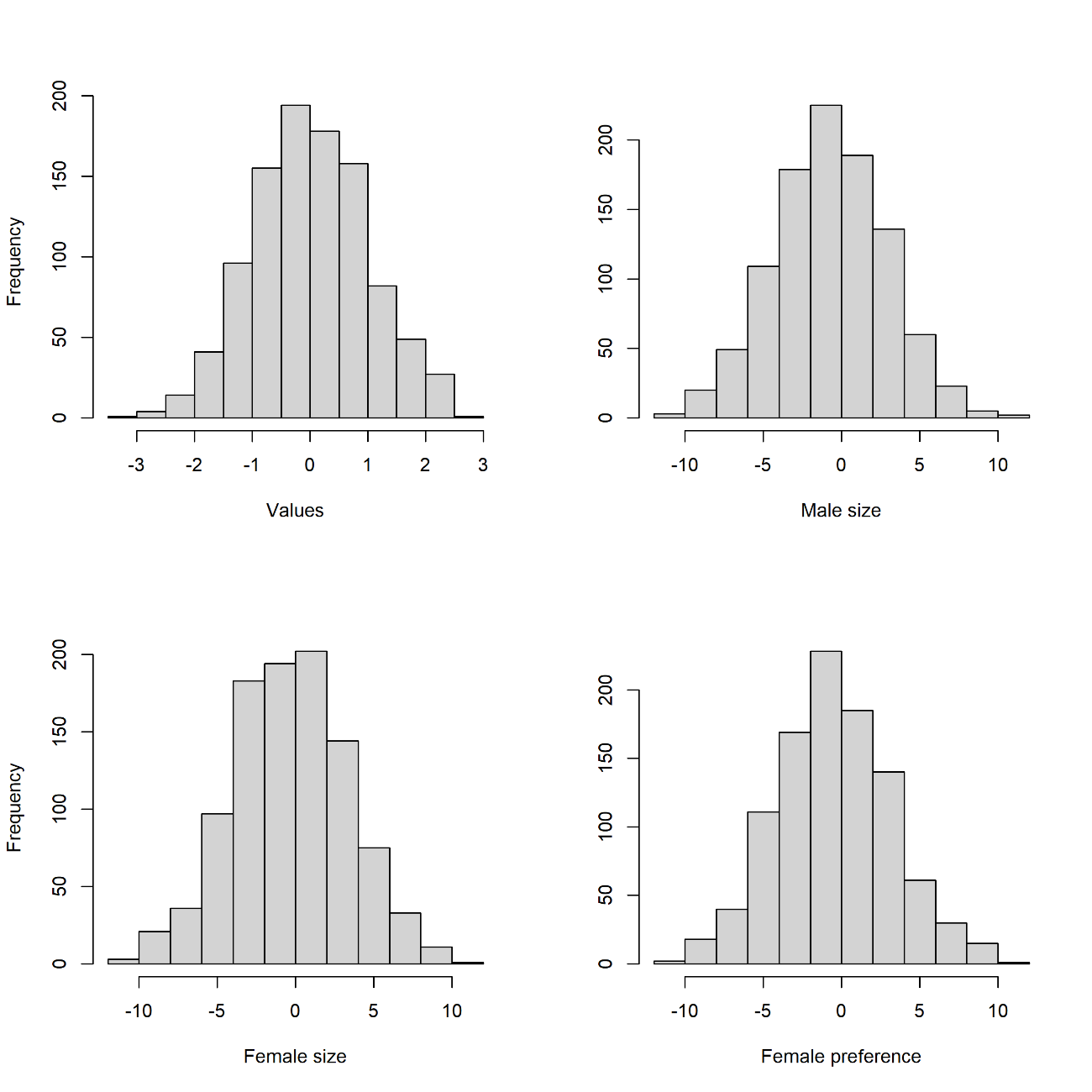


**D**

**C**

**B**

**A**

Fig S10 Initial values simulated for *Hasarius adansoni* phenotypes for the 10-genes model. (A) A random sample from a normal distribution, for comparison. (B) Male size. (C) Female size. (D) Female preference.


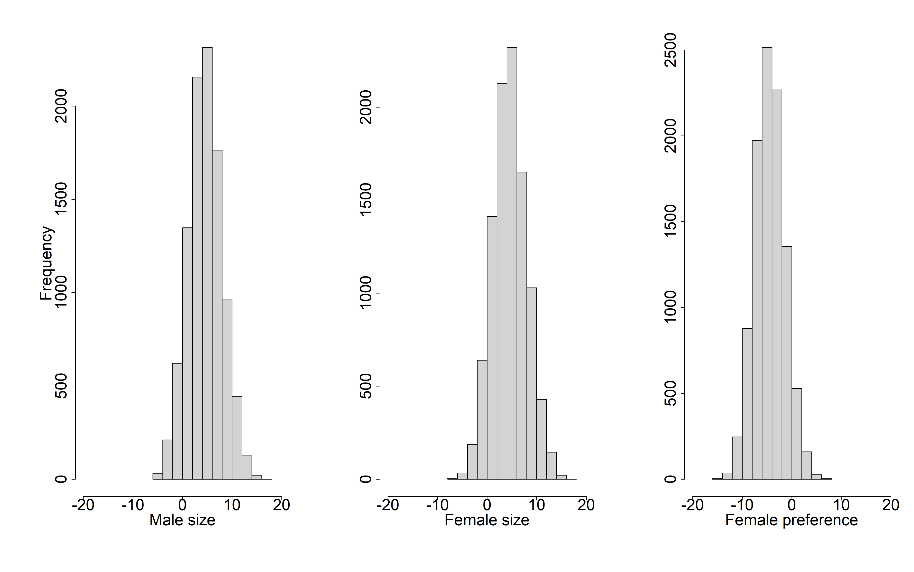

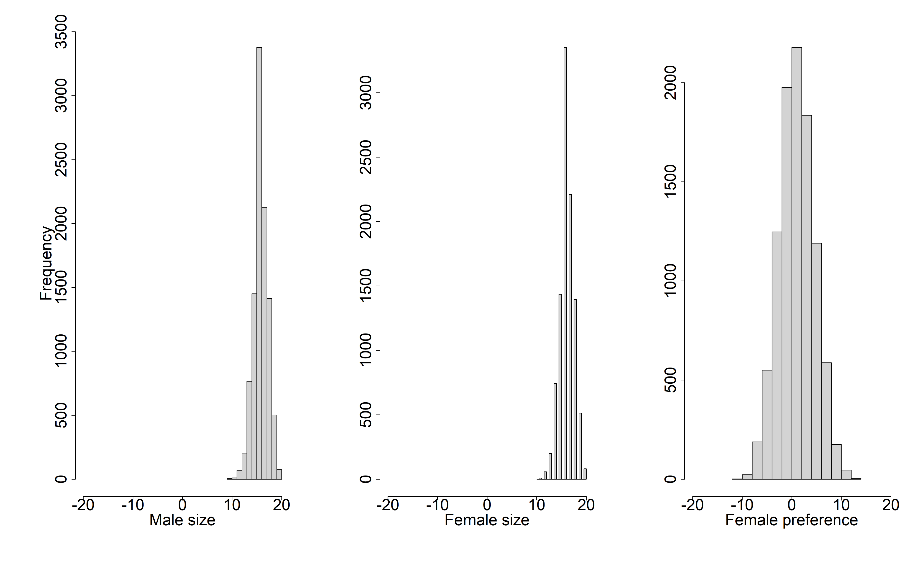

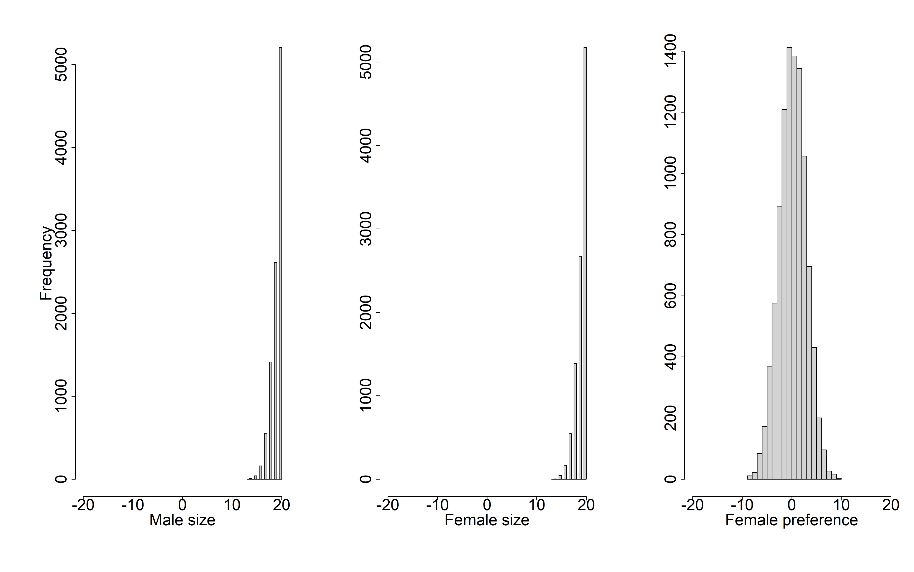

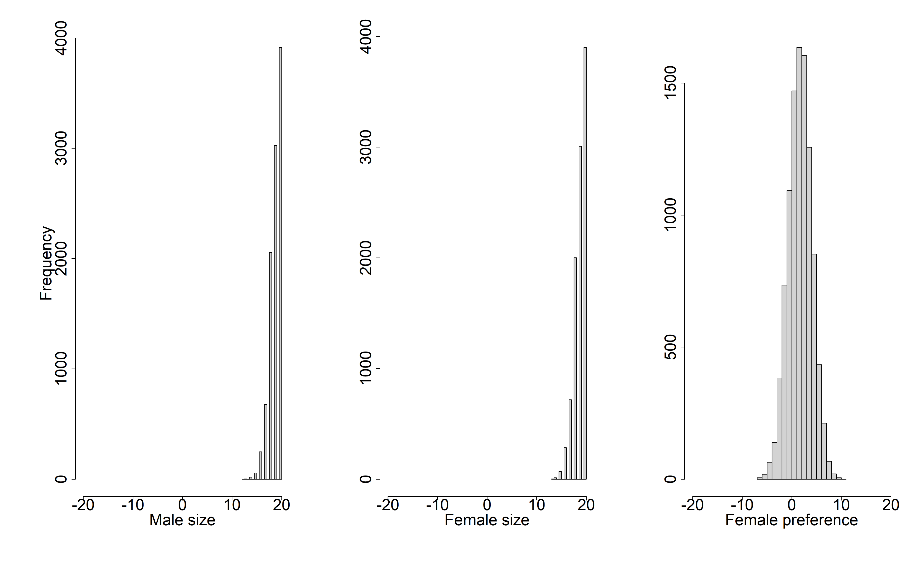


**A**

**B**

**C**

**C**

**D**

Fig S11 Evolutionary path of sizes and female preferences of *Hasarius adansoni* in the 10-genes model. The figure shows frequency distributions of all phenotypes after (A) 5 generations, (B) 25 generations, (C) 45 generations, and (D) 75 generations. Parental frequency distributions are shown in Fig S10.


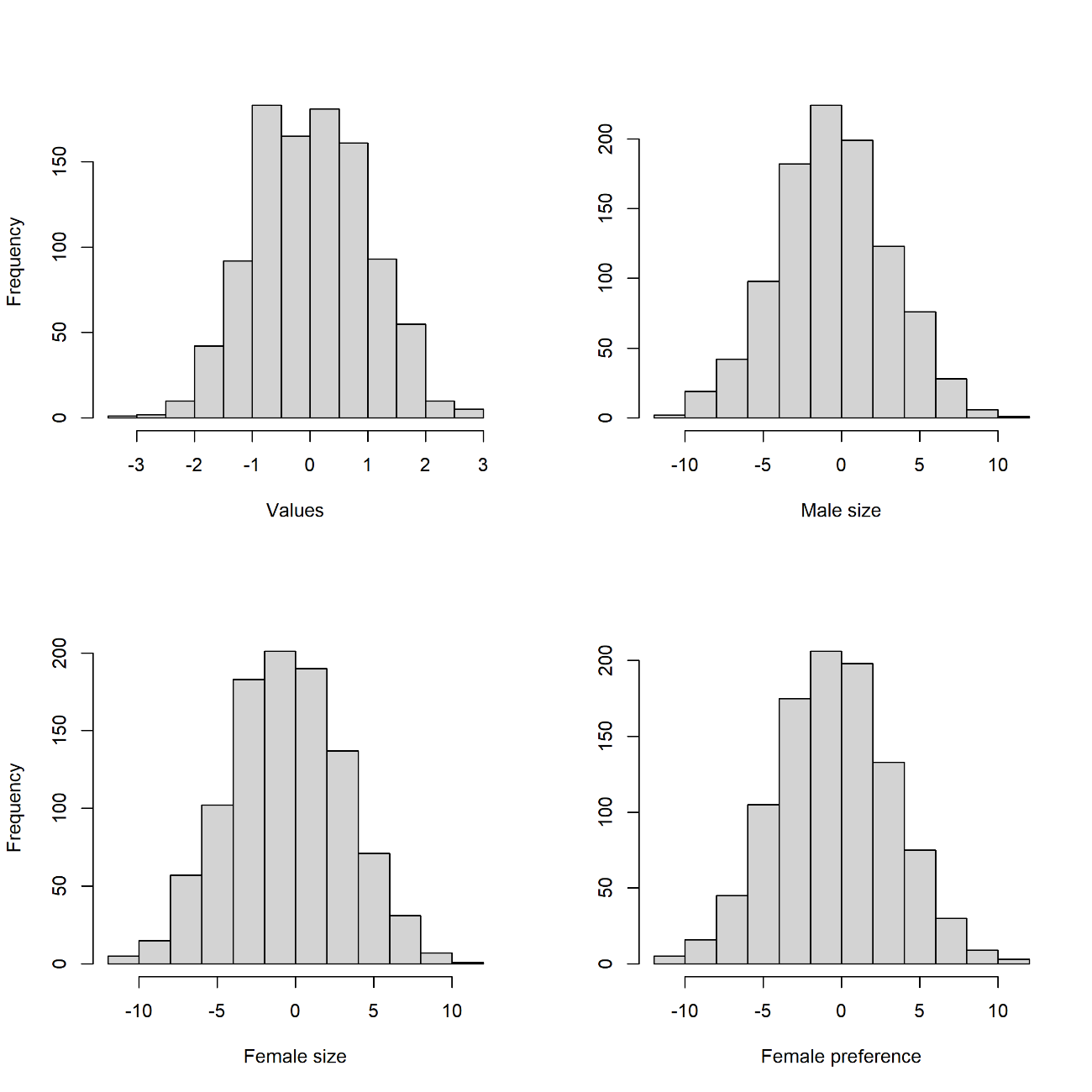


**D**

**C**

**B**

**A**

Fig S12 Initial values simulated for *Hasarius adansoni* phenotypes for the 10-genes+environment model. (A) A random sample from a normal distribution, for comparison. (B) Male size. (C) Female size. (D) Female preference.


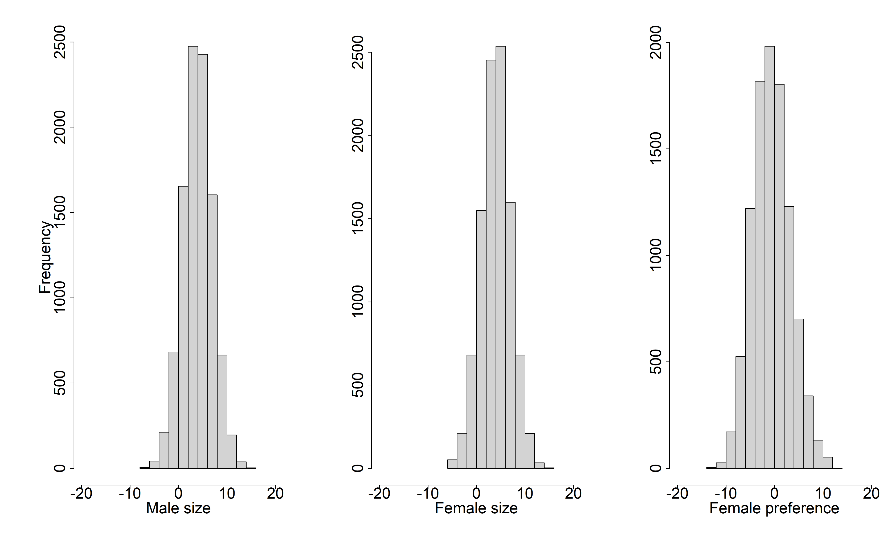

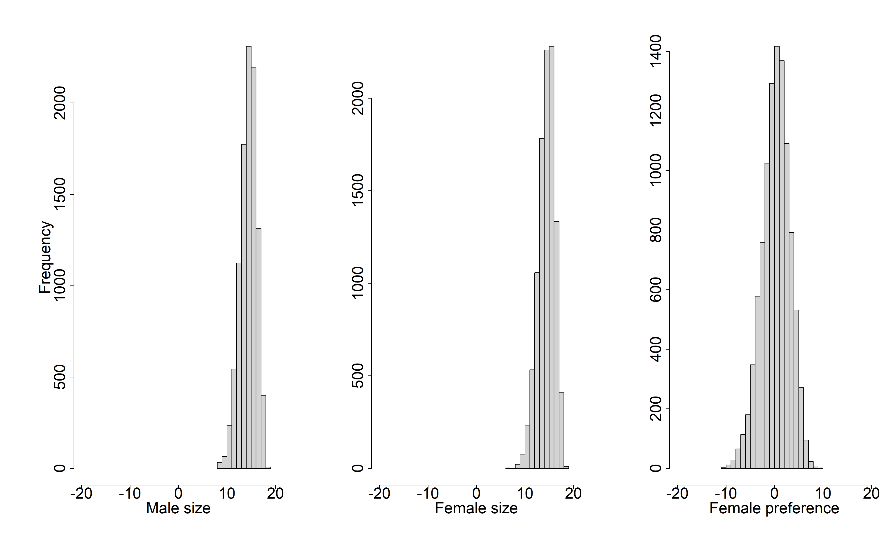

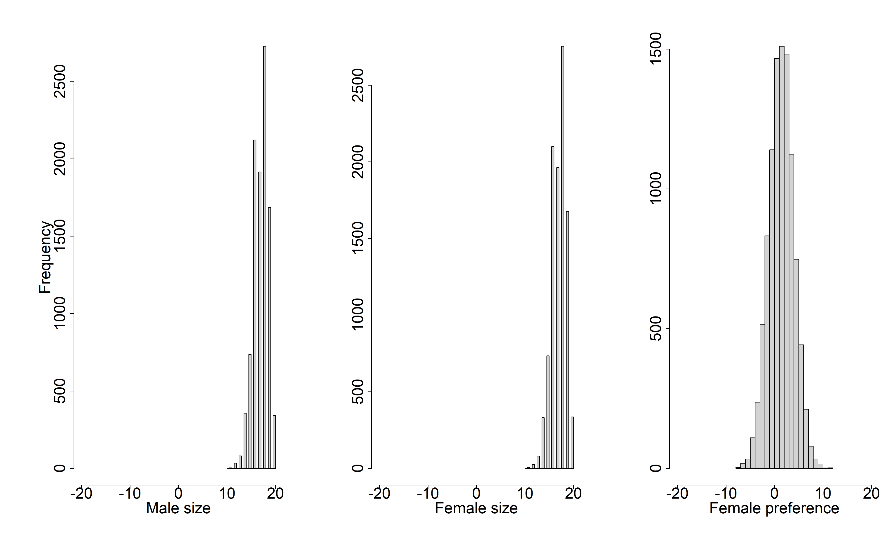

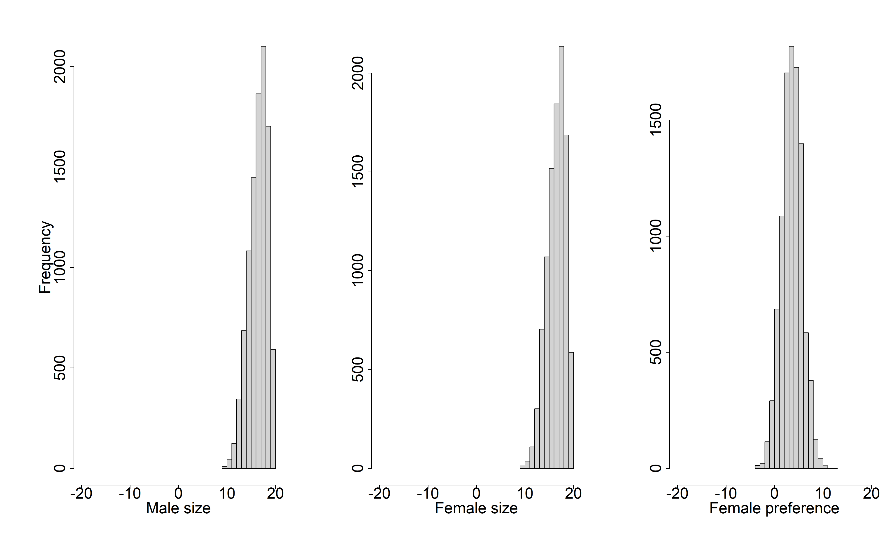


**A**

**B**

**C**

**D**

Fig S13 Evolutionary path of sizes and female preferences of *Hasarius adansoni* in the 10-genes + environment model. The figure shows frequency distributions of all phenotypes after (A) 5 generations, (B) 25 generations, (C) 45 generations, and (D) 75 generations. Parental frequency distributions are shown in Fig S12.


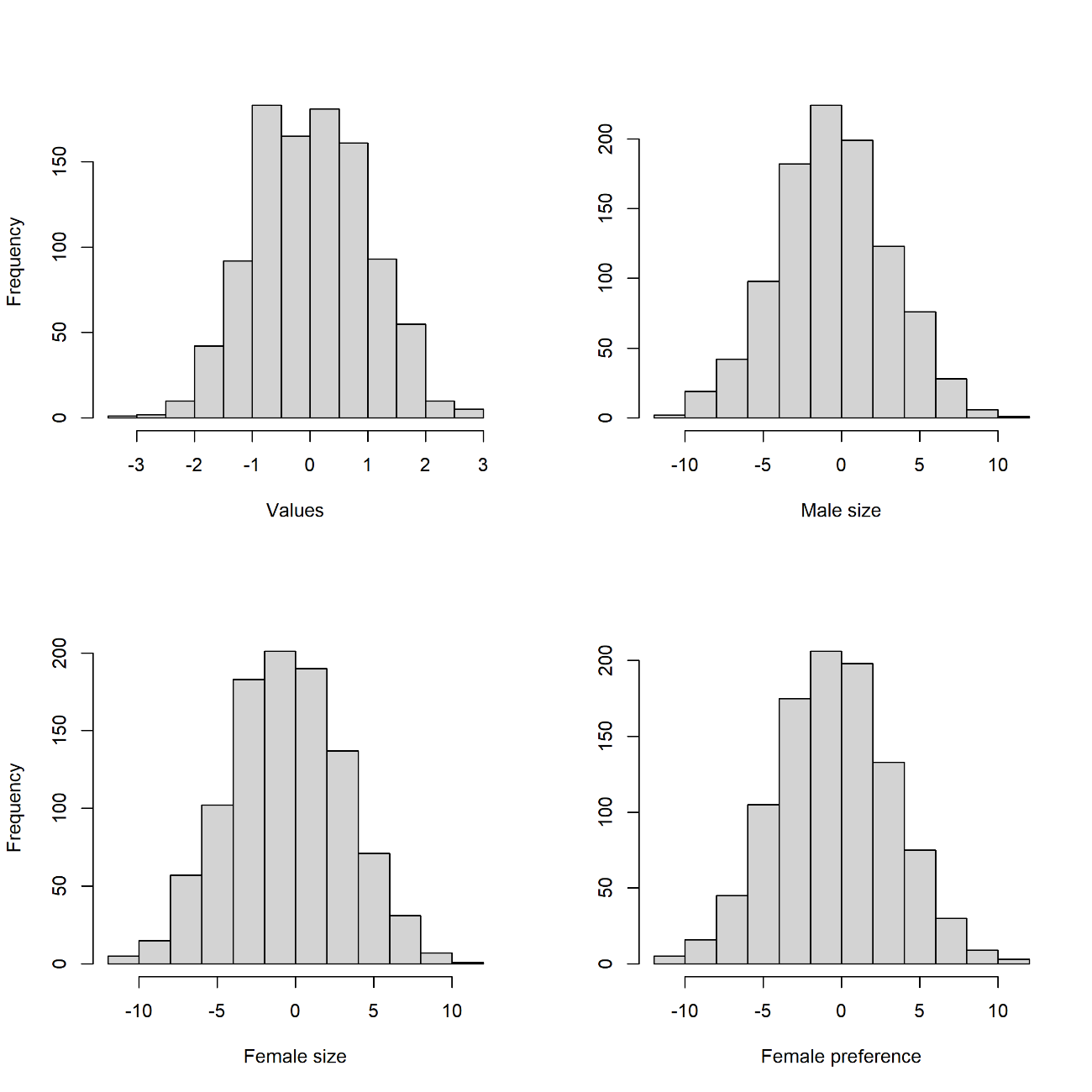


**D**

**C**

**B**

**A**

Fig S14 Initial values simulated for *Hasarius adansoni* phenotypes for the 10-genes+overlap model. (A) A random sample from a normal distribution, for comparison. (B) Male size. (C) Female size. (D) Female preference.

**A**


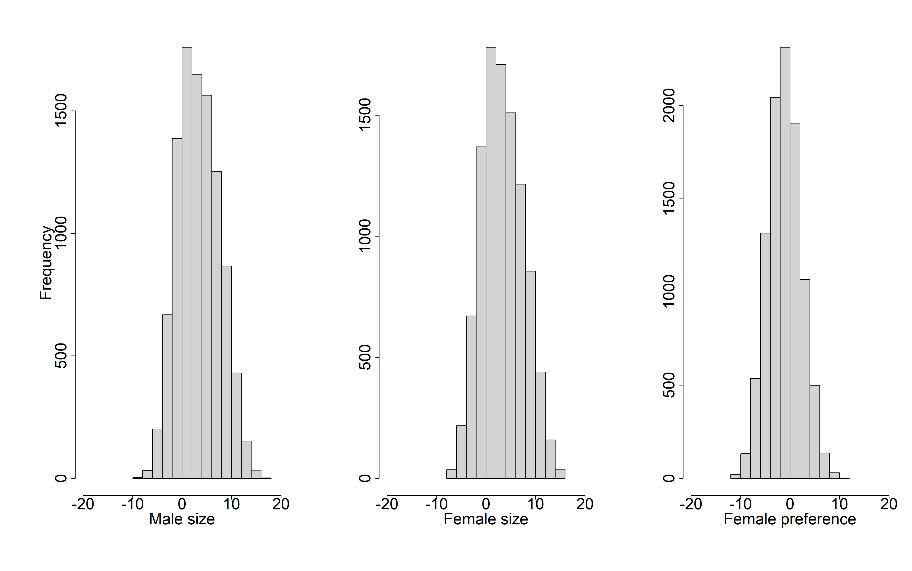

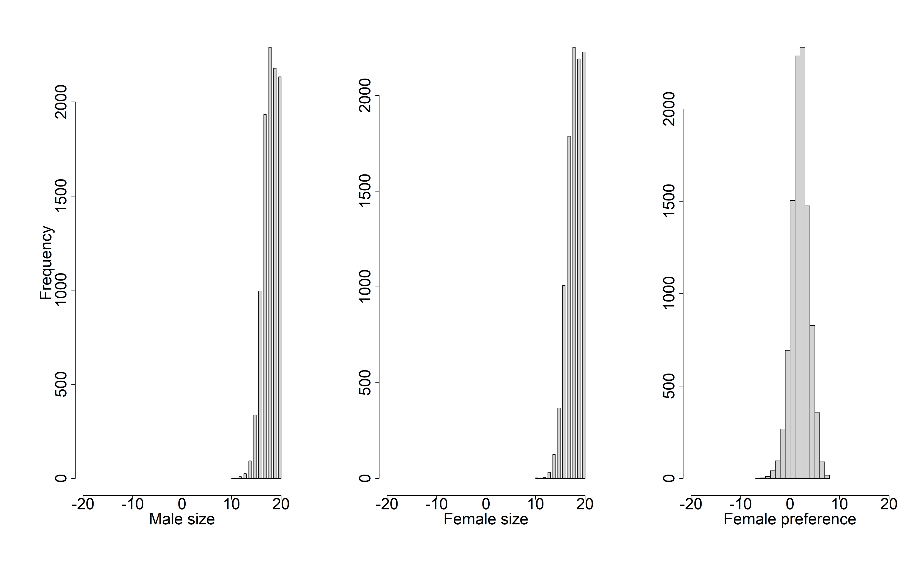

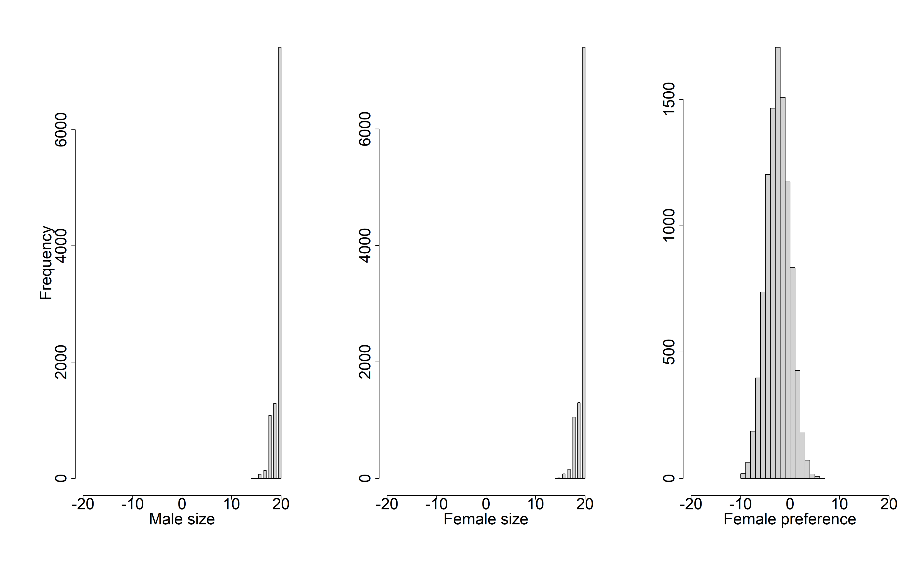

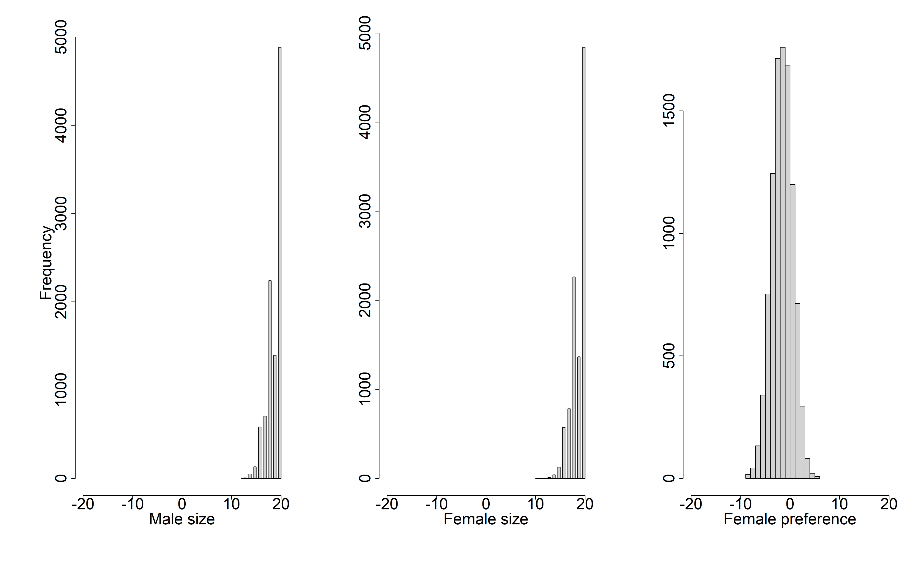


**B**

**C**

**D**

Fig S15 Evolutionary path of sizes and female preferences of *Hasarius adansoni* in the 10-genes+overlap model. The figure shows frequency distributions of all phenotypes after (A) 5 generations, (B) 25 generations, (C) 45 generations, and (D) 75 generations. Parental frequency distributions are shown in Fig S14.


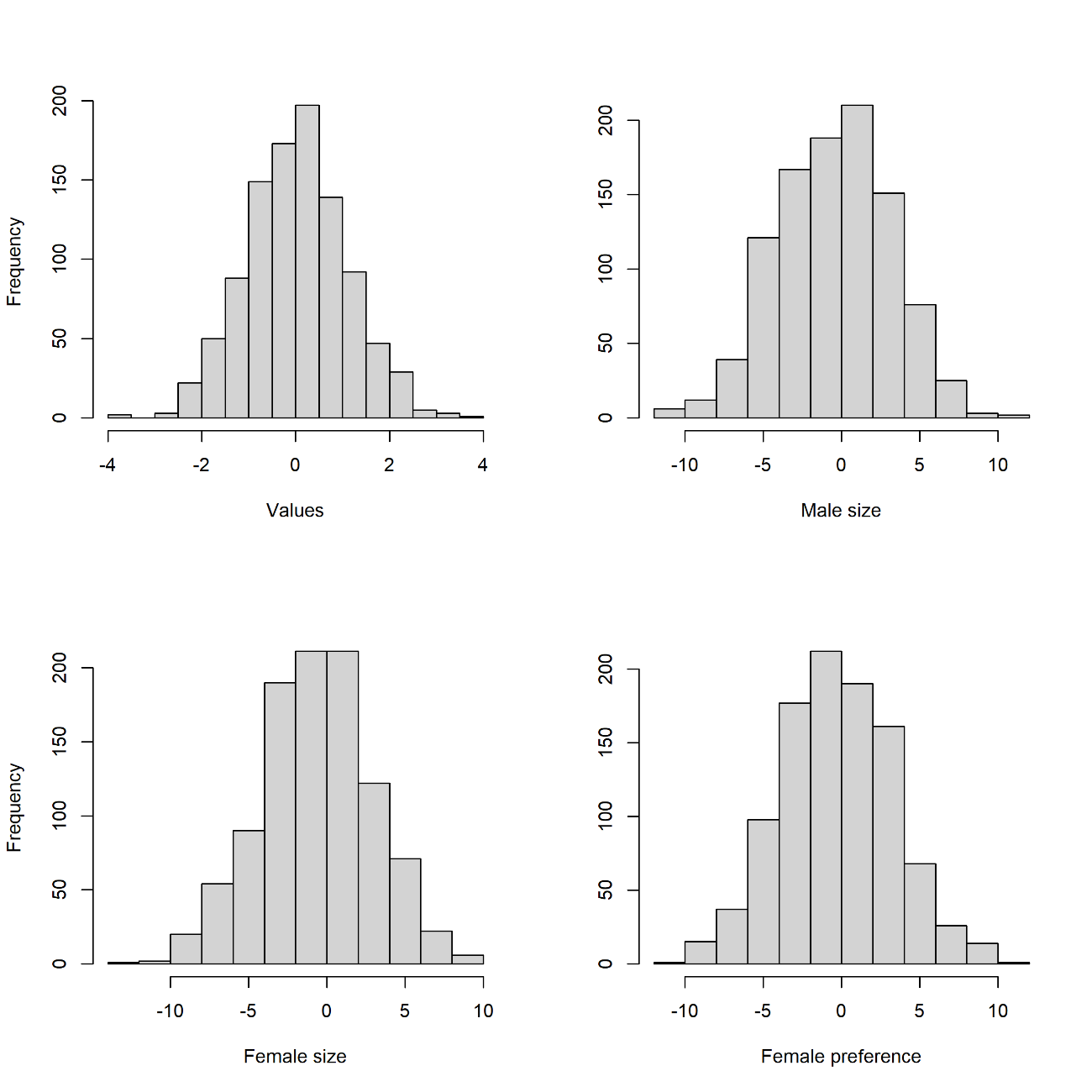


**D**

**C**

**B**

**A**

Fig S16 Initial values simulated for *Hasarius adansoni* phenotypes for the 10-genes+environment+overlap model. (A) A random sample from a normal distribution, for comparison. (B) Male size. (C) Female size. (D) Female preference.


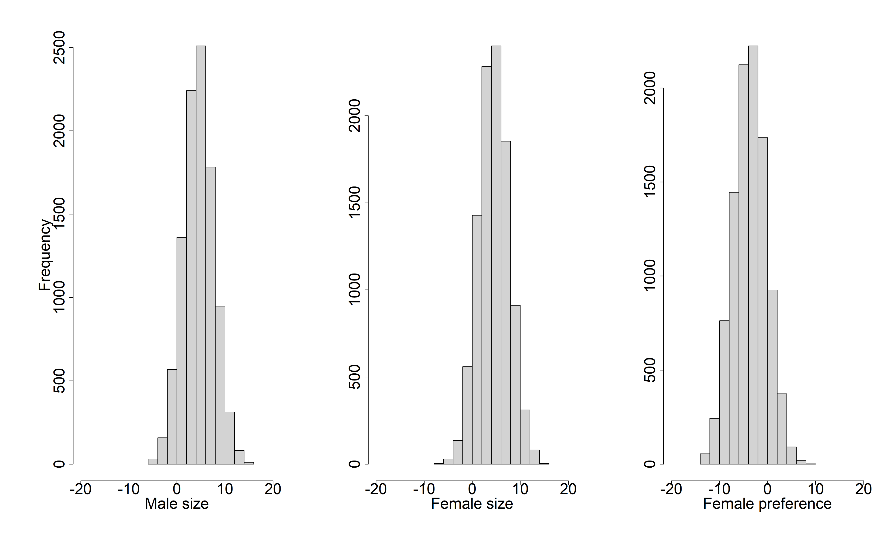

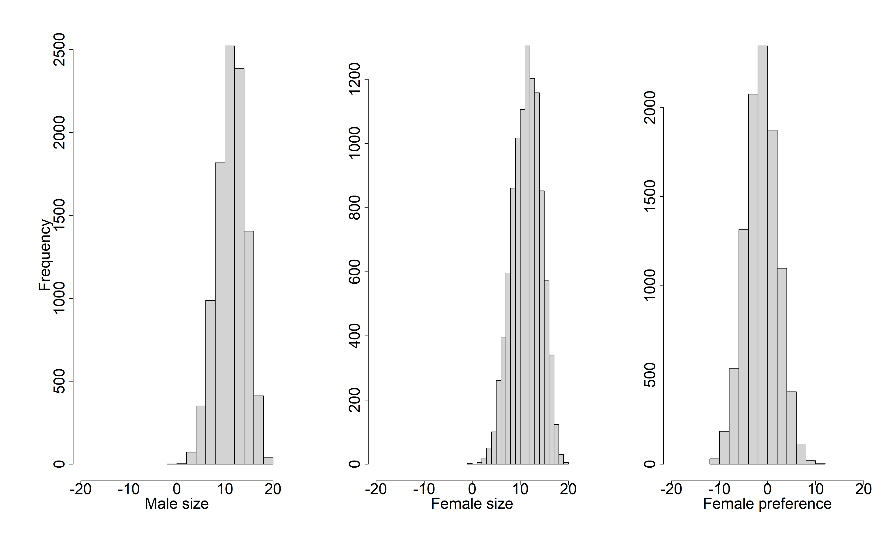

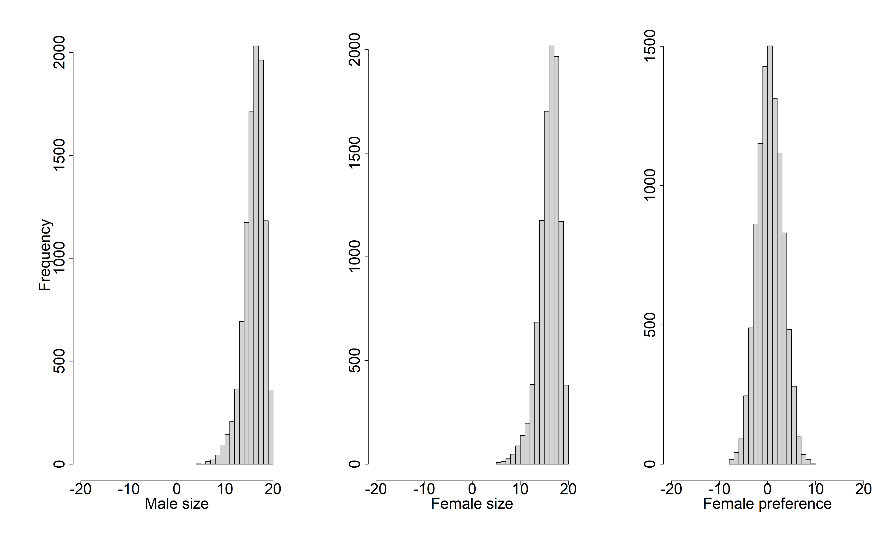

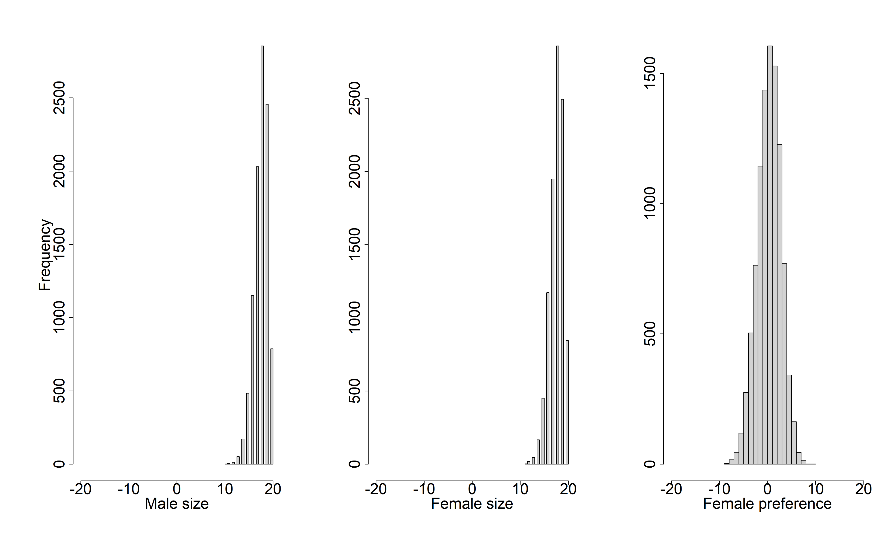


**A**

**B**

**C**

**D**

Fig S17 Evolutionary path of sizes and female preferences of *Hasarius adansoni* in the 10-genes+environment+overlap model. The figure shows frequency distributions of all phenotypes after (A) 5 generations, (B) 25 generations, (C) 45 generations, and (D) 75 generations. Parental frequency distributions are shown in Fig S16.


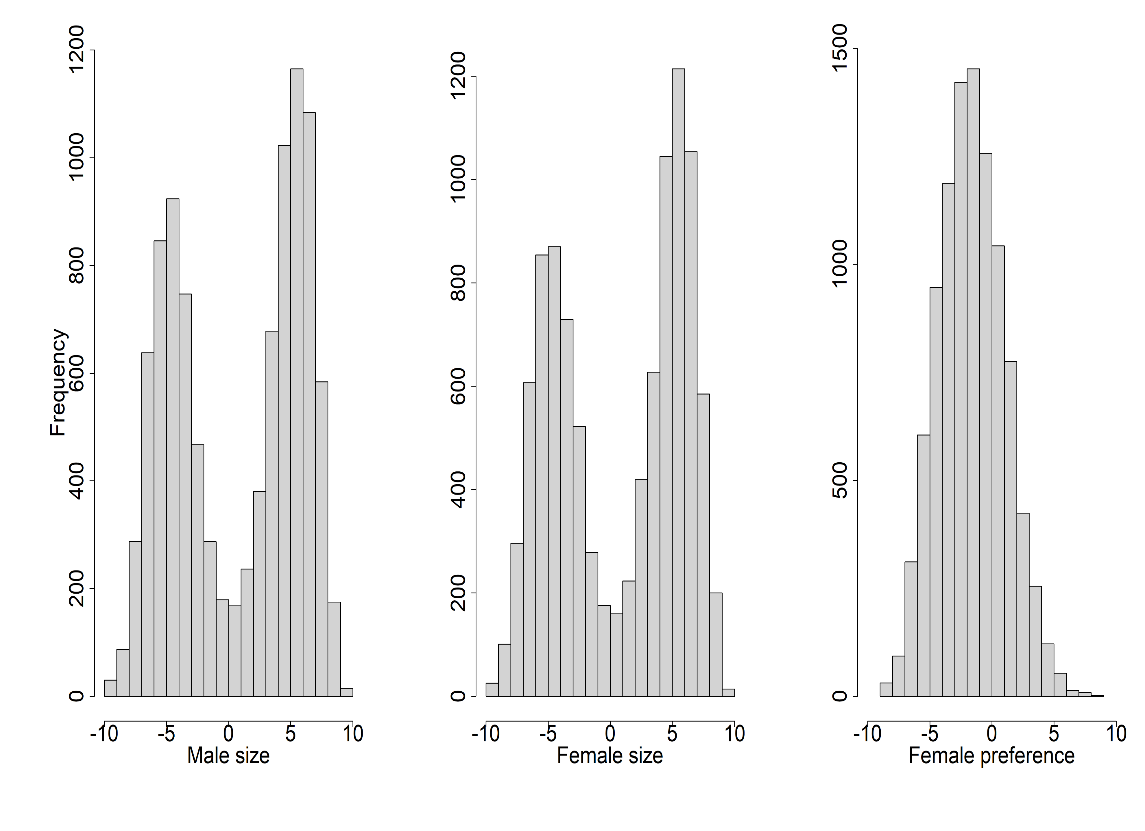

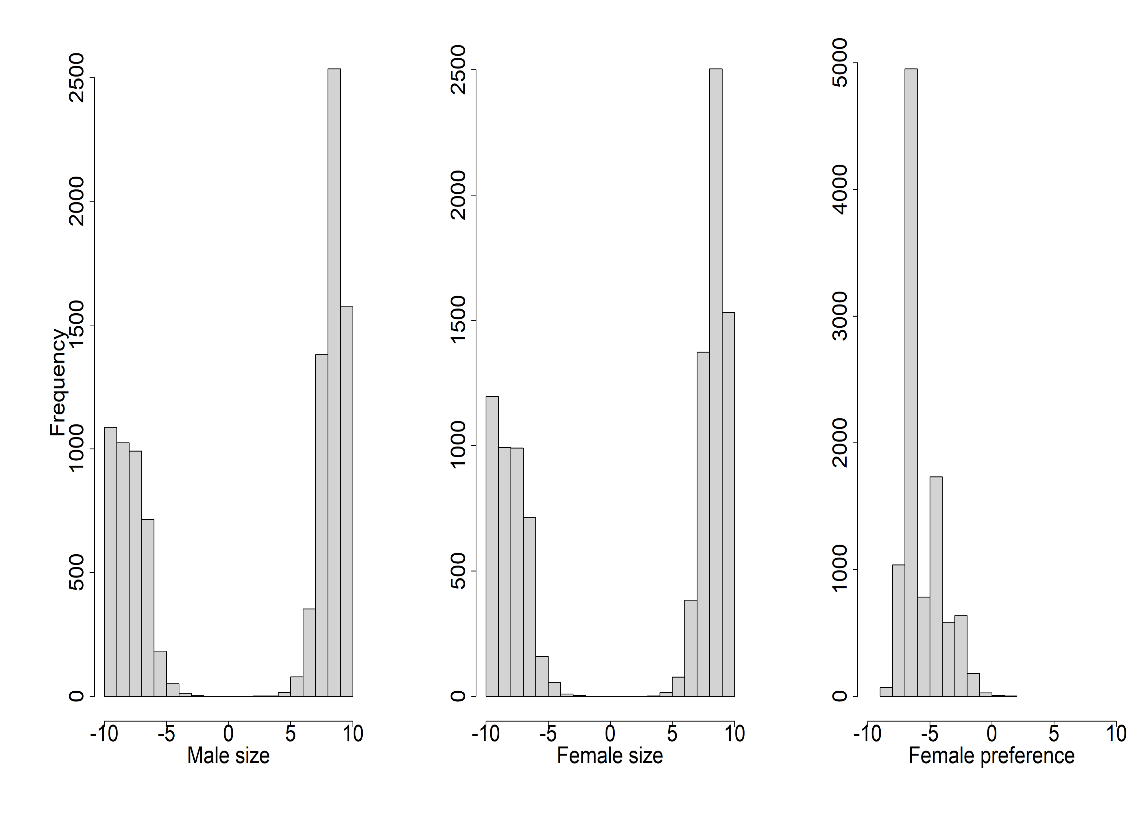


**B**


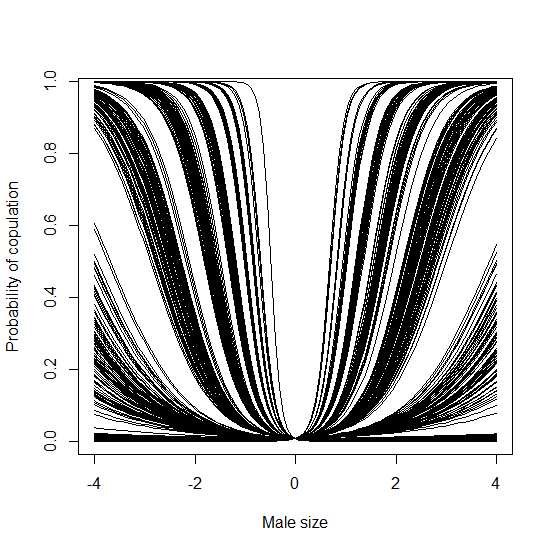


**A**

FigS18 Results of the 5-genes model when every female’s *α_i_* is set to -5, and their *β_i_* are conditional on their size, generating assortative mating for size. (A) Probability of a female mating in relation to a male size. Each line represents a different female from the initial population of 1000 females. (B) Male and female sizes after 5 and 20 generations, showing clear speciation. Parental size distributions are similar to other models. See main text for more details
